# Anesthetic propofol enhances cisplatin-sensitivity of non-small cell lung cancer cells through N6-methyladenosine-dependently regulating the miR-486-5p/RAP1-NF-κB axis

**DOI:** 10.1186/s12885-022-09848-y

**Published:** 2022-07-14

**Authors:** Quan Ling, Shaoyong Wu, Xiaozu Liao, Chiyi Liu, Yong Chen

**Affiliations:** 1 The First Department of Anesthesia, Zhongshan City People’s Hospital, No.2, Sunwen East Road, Shiqi District, Zhongshan, 528400 China; 2 Department of Anesthesiology, cancer prevention and treatment center, Sun Yat Sen University, Guangzhou, 510060 China

**Keywords:** Propofol, Non-small cell lung cancer, Cisplatin resistance, miR-486-5p, Ras-associated protein1, NF-kappaB

## Abstract

**Background:**

Drug resistance is a considerable challenge for chemotherapy in non-small cell lung cancer (NSCLC). Propofol, a commonly used intravenous anesthetics, has been reported to suppress the malignancy of various cancers. However, the effects of propofol on cisplatin (DDP) sensitivity in NSCLC and its molecular mechanisms have not been clearly clarified yet, and the present study aimed to resolve this problem.

**Methods:**

NSCLC cells were co-treated with propofol and DDP, Cell Counting kit-8 assay, colony formation assay and flow cytometry were conducted to test the role of propofol in regulating DDP-resistance in NSCLC. Next, through conducting quantitative real-time polymerase chain reaction, dual-luciferase gene reporter system and western blot, the responsible molecular axis in propofol regulating the DDP sensitivity in NSCLC was uncovered, and the function verification experiments were performed by transfection with the inhibitors or small interfering RNAs of those molecules.

**Results:**

Propofol suppressed cell viability, colony formation ability, tumorigenesis, and promoted cell apoptosis to enhance DDP-sensitivity in NSCLC *in vitro* and *in vivo*. Propofol increased miR-486-5p level in NSCLC cells and xenograft tumors tissues in a N6-methyladenosine (m6A)-dependent manner, thus inactivating the Ras-associated protein1 (RAP1)-NF-kappaB (NF-κB) axis. Propofol regulated the miR-486-5p/RAP1-NF-κB axis to improve DDP-sensitivity in NSCLC.

**Conclusions:**

Taken together, this study firstly investigates the detailed molecular mechanisms by which propofol enhanced DDP-sensitivity in NSCLC cells, and a novel m6A-dependent miR-486-5p/RAP1-NF-κB axis is identified to be closely associated with the process.

**Supplementary Information:**

The online version contains supplementary material available at 10.1186/s12885-022-09848-y.

## Introduction

Lung cancer is a worldwide popular malignant tumor, and over 1 million people died of it every year [1]. Non-small cell lung cancer(NSCLC)is the most common histological type of lung cancer in clinic, accounting for about 80% [2]. Most of the patients have been in an advanced stage at the time of diagnosis, and platinum-based chemotherapy is the standard treatment for these patients. Unfortunately, acquired resistance is a big challenge during chemotherapy, leading to therapy failure and a poor prognosis [3–5]. Although the drug resistance in NSCLC has been studied for several years, the mechanisms have not been fully uncovered, and there is still a lack of effective improvement strategies. Propofol is a short-acting intravenous anesthetic, used for the induction and maintenance of general anesthesia. Recently, a growing number of evidence shows that propofol affects cancer development, such as the suppression of tumor growth, migration and drug resistance [6–8]. Consequently, propofol is regarded to be a better choice for cancer surgery. In NSCLC, propofol has also been reported to enhance the sensitivity to cisplatin [9]. However, the molecular mechanism behind the specific effects of propofol in NSCLC remains largely unknown.

MicroRNAs(miRNAs) are a series of small, single-strand, noncoding RNA with a length of 21 ~ 23 nucleotides. miRNA involves in various biological processes of cells, including proliferation, migration, invasion, and apoptosis. Therefore, its role in the cancer is attracting growing attention. miR-486 has been considered as a tumor-suppressive miRNA in cancers [10–12]. Previous studies demonstrates that miR-486 also inhibits cell proliferation, invasion and tumorgenesis through different pathway in NSCLC [13–15]. In addition, the miR-486 expression relates to the cisplatin sensitivity in several cancers, including NSCLC, and upregulation of miR-486 is reported to enhance the cisplatin sensitivity [16–19]. One study by *Yang N *et al. [20] reports that propofol inhibits lung cancer cell viability and induces cell apoptosis by upregulating miR-486 expression. However, it is still unclear whether propofol reduces ciaplatin resistance of NSCLC via miR-486.

Ras-associated protein1 (RAP1) is a member of RAS oncogene family. RAP1 supports cancer progression through various oncogenic events, such as promotion of cell proliferation and mediating chemical resistance [21]. RAP1 is an essential modulator of the NF-kappaB (NF-κB) pathway mediated inflammation [22, 23]. A study by Ryan, S. L et al. [24] suggests that small molecule inhibitor of NF-κB re-sensitized cisplatin-resistant NSCLC cells. RAP1 forms a complex with IkappaB kinases (IKKs) and promoting activation of the NF-κB pathway [25]. RAP1-NF-κB is also reported to be the key pathway of cisplatin resistance in NSCLC [26]. MiRNAs usually negatively regulate the gene expression, via binding to the 3’ untranslated region of the target mRNAs and resulting in its degradation. In our study, we identified the RAP1-NF-κB axis as the target of miR-486-5p in regulating the cisplatin resistance of NSCLC, and miR-486-5p levels could be elevated by propofol. Mechanismly, propofol enhances cisplatin sensitivity of NSCLC through miR-486-5p/RAP1-NF-κB axis. The finding provides a deeper understanding about the molecular mechanism of propofol in the platinum resistance of NSCLC, and might support the application of propofol in NSCLC chemotherapy.

## Materials and methods

### Cells culture

Lung adenocarcinoma cell line A549 and its cisplatin (DDP)-resistant cell A549/DDP, lung squamous cell carcinoma cell line SKMES and its DDP-resistant cell SKMES/DDP, and human embryonic kidney cell line HEK293 were bought from Biovector Science Lab. All cells were cultured in Dulbecco’s modified Eagle medium (DMEM, ThermoFisher, Shanghai, China) supplemented with 10% fetal bovine serum (FBS, Sigma, St. Louis, Missouri, USA) and 1% double antibiotics (penicillin and streptomycin, Invitrogen, Carlsbad, CA, USA). The mediums were all maintained in an incubator containing 5% CO_2_ at 37 °C.

### Cell transfection

Negative control (NC) mimic, miR-486-5p mimic, NC inhibitor, miR-486-5p inhibitor, siRNA-NC and siRNA-RAP1 were synthesized by Sangon Biotech (Shanghai, China). Then they were transfected into indicated cells by using Lipofectamine 2000 Transfection Reagent (Invitrogen, Carlsbad, CA, USA) according to the manufacturer’s instruction. 48 h after transfection, the transfection efficiency was tested by qRT-PCR.

### Drug intervention

The propofol concentration used in the cells were conferred to the previous reporters [9, 20]. The above cells were all treated with low concentration of propofol (5 μg/mL) or high concentration of propofol (10 μg/mL), and stimulated with different concentrations of DDP (0 μM ~ 100 μM) for 48 h.

### Cell Counting Kit-8 (CCK-8) assay

The cell viability was evaluated through Cell Counting Kit-8 (CCK-8, Yeasen, Shanghai, China) according to the manufacturer's instructions. Cells were seeded into a 96-well plate overnight (1 × 10^4^ cells/well), and incubated for 24 h, 48 h, 72 h and 96 h respectively. At different time points, the cells were added with 10μL CCK-8 solution, and incubated for 1 h. The absorbance (OD) value at 450 nm was determined by a microplate reader (BioTek, Vermont, USA).

### Colony formation assay

The cells in logarithmic growth stage were seeded into 6-well plates (200 cells/well), and cultured for 2 weeks. The cells were fixed by 4% paraformaldehyde for 10 min, and stained by Giemsa for 30 min. The cells were counted under the low-magnification microscope.

### Flow cytometry

Cell apoptosis was detected through flow cytometry analysis. The Annexin V-FITC/PI apoptosis detection kit (Beyotime, Shanghai, China) was used to stain the cells. Briefly, cells were plated into 6-well plate (1 × 10^5^ cells/well) and correspondingly treated with propofol or DDP. Then cells were resuspended in Annexin V/FITC and PI buffer according to the manufacturer's instructions. The cells were finally analyzed by a flow cytometer (Beckman Coulter, Bria, CA, USA).

### Xenograft experiments

BALB/c nude mice (female, 4–5 weeks old, specific pathogen free animals) were purchased from the Saiye Model Biology Research Center (License: #SCXK(Su)2018–0003). BALB/c nude mice were divided into 4 groups, NC, DDP, Propofol and DDP + Propofol, 3 mice in each group. The method for constructing xenograft was as described in previous studied [2, 27]. In brief, A549, A549/DDP or SKMES/DDP cells (5 × 10^6^) were injected subcutaneously to nude mice. 7 days after injection, these mice were daily injected intraperitoneally with propofol (dissolved in soybean oil, 35 mg/kg) or with equal volume of soybean oil for 3 weeks, and some of them were intraperitoneally administered with DDP (5 mg/kg) every week for 3 weeks. The tumor length and width were measured every 3 days, and the tumor volume was estimated according to the formula “volume = 1/2 × length × width^2^”. After 28 days of modeling, the mice were sacrificed, and the xenografts tumors was removed and weighted. The tumor tissues of mice were then subjected to TUNEL staining.

### TUNEL staining

The tumor tissues embedded in paraffin, sectioned and dewaxed, and incubated with proteinase K at 37℃ for 30 min. TUNEL reagent (Roche, Basel, Switzerland) was prepared according to the manufacturer’s instruction, and then the section was incubated with the fresh TUNEL reaction solution at 37℃ for 1 h. After colored by DAB solution at 25℃ for 10 min, the section was counterstained with hematoxylin for 10 s. Finally, the TUNEL-positive cells were counted under a microscope (Olympus, Tokyo, Japan).

### Quantitative real-time polymerase chain reaction (qRT-PCR)

Total RNA was extracted from tissues or cells using TRIzol reagent (Beyotime, Shanghai, China). Total RNA was reverse transcribed to cDNA using Taqman MicroRNA Reverse Transcription Kit or TaqMan™ Reverse transcription reagent (ThermoFisher, Shanghai, China). Subsequently, cDNA was as a template for quantitative PCR following the manufacturer’s protocol of SYBR PrimeScript RT-PCR Kit (TaKaRa, Kyoto, Japan). ABI 7500 Real-Time PCR System (USA, Carlsbad, CA, USA) was used to perform PCR process. GAPDH or U6 was considered as the internal reference, and the relative expression was calculated through 2^−△△Ct^ method. The primers were listed as following: RAP1: 5′-TGAAGGACCGCTACCTCAAG-3′(forward) and 5′-GGCTTCCACAAGCATCTTTTTG-3′(reverse); GAPDH: 5′-ACAGTCCATGCCATCACTGCC-3′ (forward) and 5′-GCCTGCTTCACCACCTTCTTG-3′ (reverse); miR-486-5p: 5′-TCCTGTACTGAGCTGCCC -3′(forward) and 5′-GTGCAGGGTCCGAGGT-3′(reverse); U6: 5′-GCTTCGGCAGCACATATACTAAA-3′(forward) and5′-GCTTCACGAATTTGCGTGTCAT-3′(reverse); Bax: 5′-TGGCCTCCTTTCCTACTTCG-3′(forward) and5′-AAATGCCTTTCCCCGTTCCC-3′(reverse); Bcl2: 5′- ACCCCTTCATCCAAGAATGCAA-3′(forward) and5′-TCTCCCGGTTATCATACCCT-3′(reverse);

Caspase3: 5′- TCATGCACATCCTCACTCGT-3′(forward) and 5′-AAACATGCCCCTACCCCACT-3′(reverse).

### Western blot

Total protein was released from tissues and cells by RIPA lysis buffer (Beyotime, Shanghai, China). The concentration of extracted protein was determined by BCA Protein quantitative Kit (Beyotime, Shanghai, China). The electrophoresis and transferring to PVDF membrane were performed in a Bis–Tris Gel system (Bio-Rad, Hercules, CA, USA). The blots were cut prior to hybridization with antibody, and incubated overnight at 4 ℃ with primary antibodies against RAP1 (#ab175329, Abcam, Cambridge, UK), IκBα (#ab32518, Abcam), p-IκBα (#sc-52943, Santacruze, Shanghai, China), p-NFκB p65 (#ab16502, Abcam), p-NFκB p65(Ser 536) (#sc-101752, Santacruze) and GAPDH (#ab8245, Abcam). After washing for 3 times, the blots were incubated with goat anti-mouse IgG secondary antibodies (#ab97040) for 1 h at 37 °C. The proteins were visualized by using enhanced chemiluminescence kit (Beyotime), and then quantified by ImageJ Software.

### Dual luciferase reporter gene system

The binding site between miR-486-5p and RAP1 was predicted through online starbase website (https://starbase.sysu.edu.cn/agoClipRNA.php?source=mRNA). The mutant sequence around target sites of RAP1 was designed. Dual Luciferase Reporter Assay Kit (Promega, Beijing, China) was used to verify the binding relationship. The mutant sequence or wild-type sequence of RAP1 were cloned to a reporter plasmid vector. Then they were separately co-transfected into HEK293 cells with NC mimic or miR-486-5p mimic. After 48 h of transfection, the cells were lysed, and the luciferase activity was detected with a microplate reader (BioTek, Vermont, USA). The relative firefly luciferase activity was normalized to the Renilla luciferase activity.

### Statistics

All data was analyzed by GraphPad Prism version 8.0 software (GraphPad, San Diego, USA). One-way ANOVA was used to compare 3 or more groups, and then LSD method was performed for post hoc test. Independent sample t-test was used to compare 2 groups. In all cases, P value less than 0.05 was regarded as statistically significant difference.

## Results

### Propofol dose-dependently increased DDP-sensitivity in NSCLC cells

**Fig. 1:**
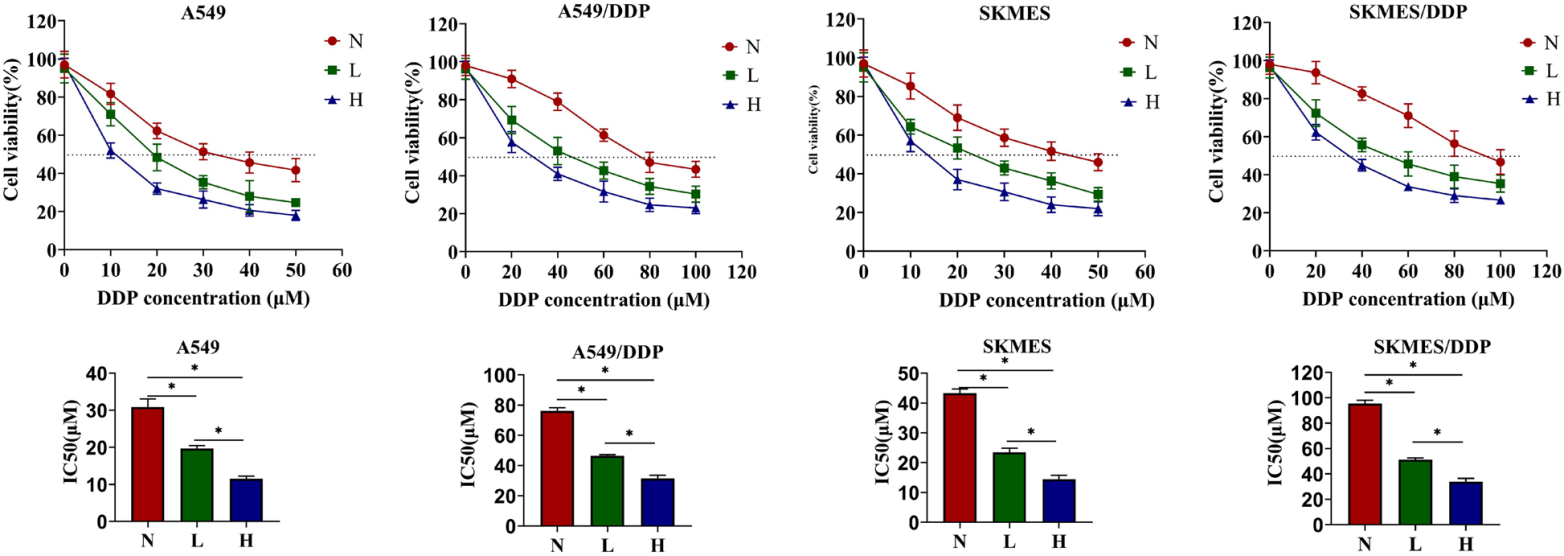
Propofol dose-dependently increased DDP-sensitivity in NSCLC cells. The DDP-sensitive NSCLC cells (A549 and SKMES) and the DDP-resistant cells (A549/DDP and SKMES/DDP) were all pretreated with low concentration of propofol (5 μg/mL) or high concentration of propofol (10 μg/mL). Then A549 and SKMES were stimulated with different concentrations of DDP (0, 10, 20, 30, 40, 50 μM) for 48 h, while A549/DDP and SKMES/DDP were stimulated with different concentrations of DDP (0, 20, 40, 60, 80, 100 μM) for 48 h. The cytotoxicity of DDP was evaluated through CCK8 assay, and the IC50 value of DDP in different cells were calculated. *P < 0.05. N, without propofol. L, low concentration of propofol. H, high concentration of propofol

Firstly, to confirm the effects of propofol on DDP-sensitivity in NSCLC, the DDP-sensitive NSCLC cell (A549 and SKMES) and the DDP-resistant NSCLC cells (A549/DDP and SKMES/DDP) were all pretreated with propofol (0, 5 or 10 μg/mL), and then administered with different concentrations of DDP (0 ~ 100 μM). CCK8 assay was conducted to evaluate the cytotoxicity of DDP in cells with different treatment. As shown in the dose survival curve in Fig. 1, cell viability under DDP treatment and the half inhibitory concentration (IC50 value) of DDP in 4 types of cells were all reduced by propofol, and propofol enhanced DDP-induced cytotoxicity in the NSCLC cells in a dose-dependent manner. The above results suggested that propofol increased DDP-sensitivity in NSCLC cells. Given that 40 μM of DDP alone had little effects on cell viability in the A549/DDP and SKMES/DDP cells, whereas DDP (40 μM) plus high-dose propofol (10 μg/mL) significantly suppressed cell viability in the DDP-resistant NSCLC cells, we selected this treatment strategy for further experiments.

### Propofol inhibited cell proliferation and promoted cell apoptosis in NSCLC cells treated with DDP

**Fig. 2:**
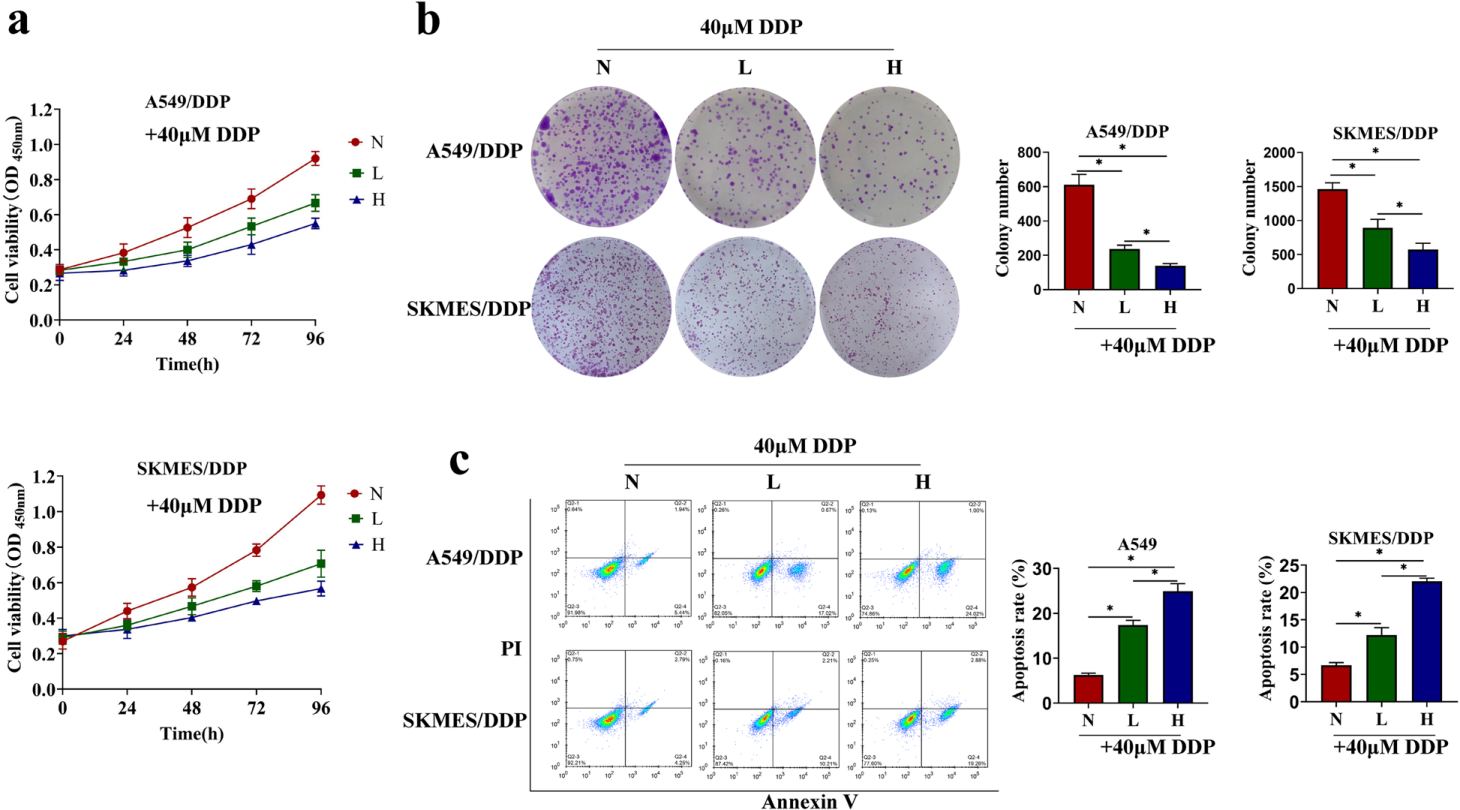
Propofol inhibited cell proliferation and promoted cell apoptosis in DDP-resistant NSCLC cells treated with DDP. A549/DDP and SKMES/DDP cells were pretreated by low concentration of propofol (5 μg/mL) or high concentration of propofol (10 μg/mL), and then treated with DDP (40 μM). **a** The cell viability at different time (0, 24, 48, 72, 96 h) was evaluated through CCK8 assay. **b** The colony formation ability was measured by Giemsa staining. **c** The apoptosis was detected by flow cytometry. *P < 0.05. N, without propofol. L, low concentration of propofol. H, high concentration of propofol

As previously described, examination of cell proliferation abilities and apoptotic cell death are two pivotal indicators to evaluate drug resistance [28], to ensure whether propofol increased DDP-sensitivity in the NSCLC cells, the DDP-resistant cells and DDP-sensitive cells were administered with DDP plus propofol (0 μg/mL, 5 μg/mL or 10 μg/mL), and CCK-8 assay (Fig. 2a, Figure S1a), colony formation assay (Fig. 2b), flow cytometry (Fig. 2c) and qRT-PCR (Figure S1b) were respectively performed to examine cell proliferation and apoptosis. The results showed that cell viability and colony formation ability in the DDP-treated A549/DDP and SKMES/DDP cells were significantly suppressed by propofol in a concentration-dependent manner (Fig. 2a-b). Meanwhile, cell viability of DDP-treated A549 and SKMES cells were further inhibited by propofol (Figure S1b). In addition, propofol also dose-dependently enhanced the promoting effects of DDP on cell apoptosis in the DDP-resistant NSCLC cells (Fig. 2c), increased pro-apoptotic genes expression (Bax and Caspase3), and reduced the anti-apoptotic Bcl-2 in the DDP-sensitive NSCLC cells (Figure S1b). These data suggested that propofol inhibited cell proliferation and promoted cell apoptosis to increase DDP toxicity in both DDP-resistant or DDP-sensitive NSCLC cells.

### Propofol enhanced the suppressive effects of DDP treatment on NSCLC cell tumorigenesis *in vivo*

**Fig. 3:**
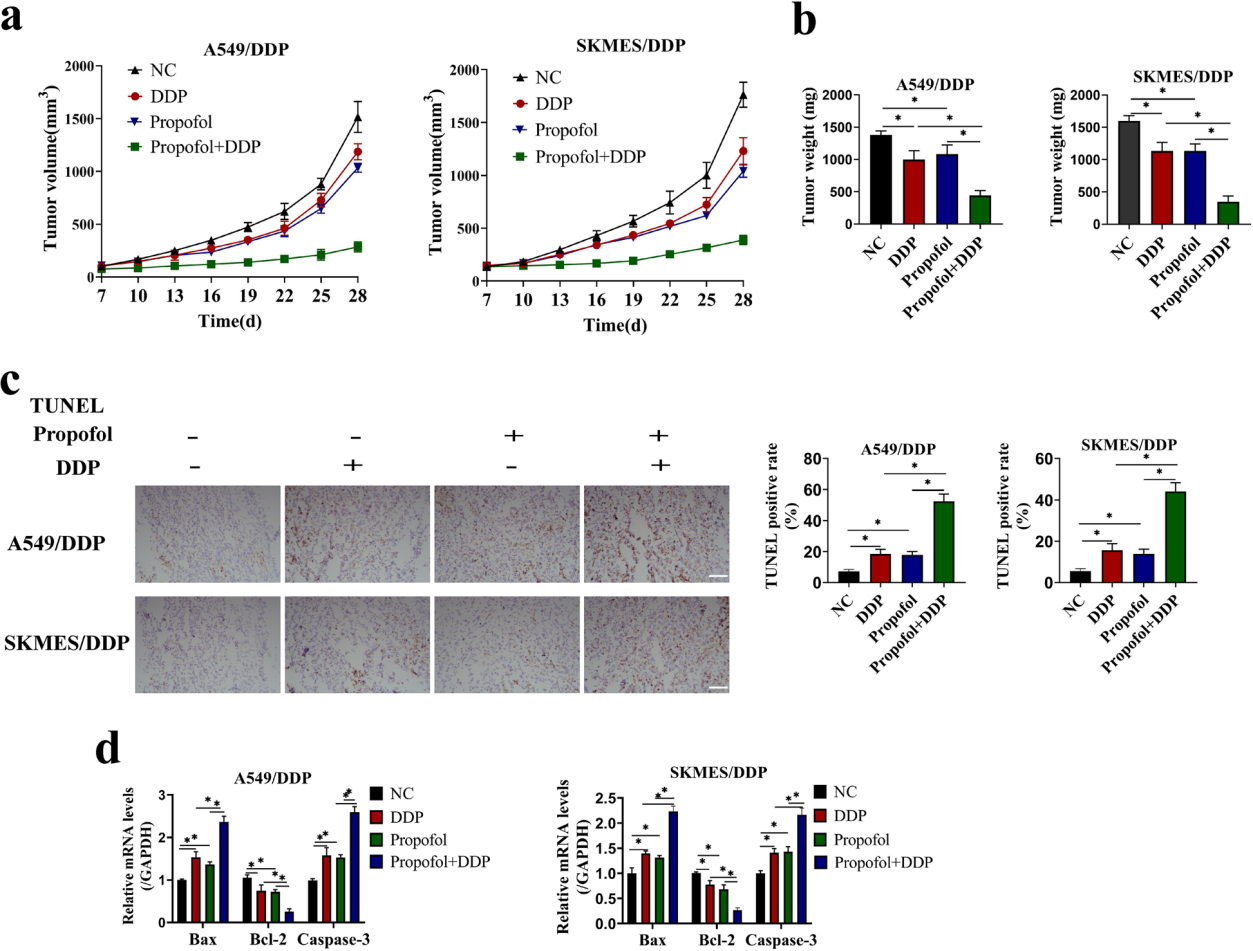
Propofol reduced DDP resistance to xenografts tumors *in vivo.* A549/DDP cells and SKMES/DDP cells were injected subcutaneously to nude mice. 7 days after injection of cells, mice were injected intraperitoneally with propofol (35 mg/kg) and DDP (5 mg/kg) for 3 weeks. **a** The tumor volume was measured every 3 days. **b** On Day 28, mice were sacrificed and the xenografts were removed and weighed. **c** The xenografts tissue was subjected to TUNEL staining and the TUNEL positive rate was regarded as apoptosis rate, and the scale bar = 100 μm. **d** The apoptosis-related genes, including Bax, Bcl-2 and Caspase-3, were measured by qRT-PCR. *P < 0.05. NC, negative control

Since the enhancing effects of propofol on DDP-sensitivity in the DDP-resistant NSCLC cells had been confirmed by *in vitro* experiment, the *in vivo* experiment was then carried out for further evidences. The two types of DDP-resistant NSCLC cells and A549 were injected subcutaneously to nude mice to construct xenografts models. Then, these mice were daily injected intraperitoneally with propofol (35 mg/kg) or the same volume of soybean oil, and administered with DDP (5 mg/kg) for 3 weeks. The results showed that propofol treatment alone inhibited tumor growth, in contrast with the negative control, and the co-treatment of propofol and DDP further enhanced the inhibitory effect of DDP on tumor growth (Fig. 3a, Figure S2a). After 28 days post-modeling, the mice were sacrificed, and the xenograft tumors was removed and weighed, and we validated that propofol also restrained tumor weight in the xenograft mice with DDP administration (Fig. 3b, Figure S2b). Then, by performing TUNEL staining assay, we found that propofol increased the TUNEL-positive apoptotic cell numbers in the DDP-treated mice tumor tissues (Figs. 3c, Figure S2c). The qRT-PCR detection of apoptosis-related genes (including Bax, BCL-2 and Caspase3) demonstrated that propofol increased the expression of pro-apoptotic Bax and Caspase3, but obviously decreased anti-apoptotic Bcl-2 levels in xenograft tumor tissues with DDP treatment (Figs. 3d, S2d). The results indicated that propofol promoted DDP-toxicity in xenografts tumors *in vivo*.

### MiR-486-5p deletion abolished the effect of propofol on NSCLC cells

**Fig. 4:**
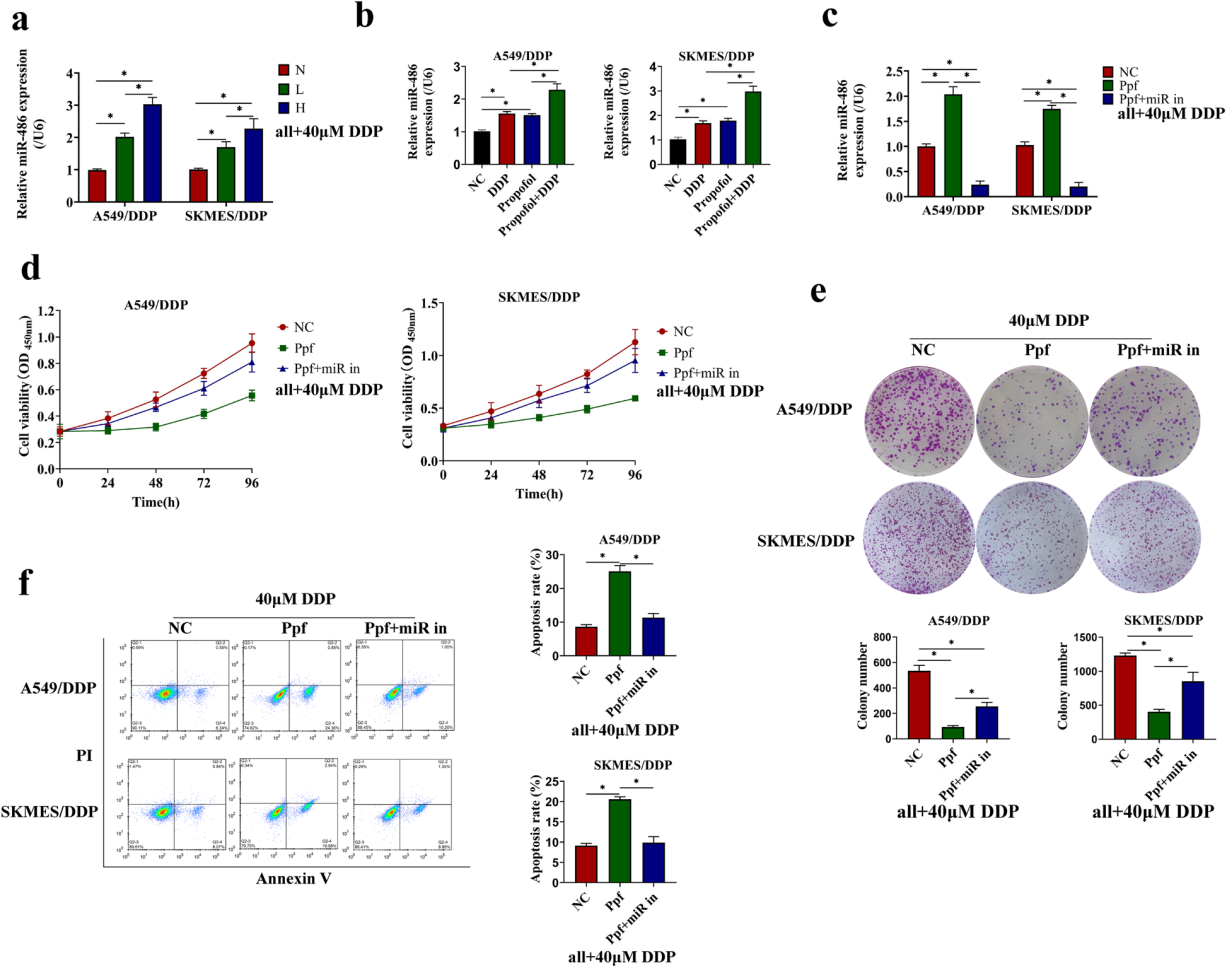
MiR-486-5p deletion abolished the effect of propofol on DDP-resistant NSCLC cells. **a**-**b** The expression of miR-486-5p in DDP-resistant cells (**a**) and in xenografts tissue (**b**) were determined by QRT-PCR. Subsequently, the A549/DDP and SKMES/DDP cells were treated with or without miR-486-5p inhibitor, and intervened with high concentration of propofol (10 μg/mL). **c** The expression of miR-486-5p was detected to test transfection efficiency. **d** Cell viability was measured by CCK-8 assay. **e** Colony formation ability was evaluated by Giemsa staining. **f** The apoptosis was detected by flow cytometry. *P < 0.05. NC, negative control. Ppf, high concentration of propofol. miR in, miR-486-5p inhibitor

Accumulating evidence suggested that downregulation of miR-486-5p contributes to the progression of NSCLC, and miR-486-5p could be a potent tumor suppressor in NSCLC [13, 15, 29–31]. To test whether propofol regulated drug resistance of NSCLC cells via miR-486-5p, the following function compensation assay was conducted. At first, we confirmed the influence of propofol on the expression of miR-486-5p by qRT-PCR, and found that propofol increased the miR-486-5p level *in vivo* and *in vitro* (Fig. 4a-b, Figure S3a-b). In addition, to investigate how propofol affected miR-486-5p expression in NSCLC, we compared the expression of the primary (pri-)miR-486 and miR-486-5p in DDP-sensitive and DDP-resistant NSCLC cells treated with or without propofol. We found that pri-miR-486 was highly expressed, while miR-486-5p was lowly expressed in A549/DDP or SKMES/DDP cells, compared to A549 or SKMES cells (Figure S4a-b). Propofol decreased the pri-miR-486 levels in NSCLC *in vivo* and *in vitro* on the basis of DDP treatment (Figure S4c-f), indicating that propofol promoted the maturation of miR-486-5p in NSCLC. Given that N6-methyladenosine (m6A) RNA modification mediated the maturation of pri-miRNAs [32, 33], we further detected the m6A enrichment in pri-miR-486 in NSCLC cells treated with or without propofol. Data showed there was higher m6A level in pri-miR-486 in DDP-sensitive cells, than that in the DDP-resistant cells, and propofol treatment significantly increased m6A enrichment in pri-miR-486 in all these NSCLC cells (Figure S4g). Knockdown of m6A methyltransferase METTL3 reversed the promoting effect of propofol on the pri-miR-486 maturation (Figure S4h-i), which proved that propofol accelerated miR-486 biogenesis through m6A modification in NSCLC.

Then, the miR-486-5p expression was knocked down in NSCLC cells by transfecting cells with miR-486-5p inhibitor (Fig. 4c, Figure S3c), and the cells were further treated with propofol and DDP. Cell proliferation and apoptosis were respectively measured, and results showed that the miR-486-5p inhibitor restored cell proliferation (Fig. 4d) and colony formation abilities (Fig. 4e), but reduced apoptosis (Fig. 4f) in the propofol and DDP co-treated A549/DDP and SKMES/DDP cells. Similarly, miR-486-5p inhibitor increased cell viability in A549 and SKMES cells co-treated by propofol and DDP (Figure S3d), decreased the levels of Bax and Caspase3 mRNA, but increased Bcl-2 mRNA expression (Figure S3e). The above data indicated that propofol upregulated miR-486-5p to enhance DDP-sensitivity in NSCLC cells in a m6A dependent manner.

### Propofol upregulated miR-486-5p to inactivate the RAP1-NF-κB pathway in NSCLC

**Fig. 5:**
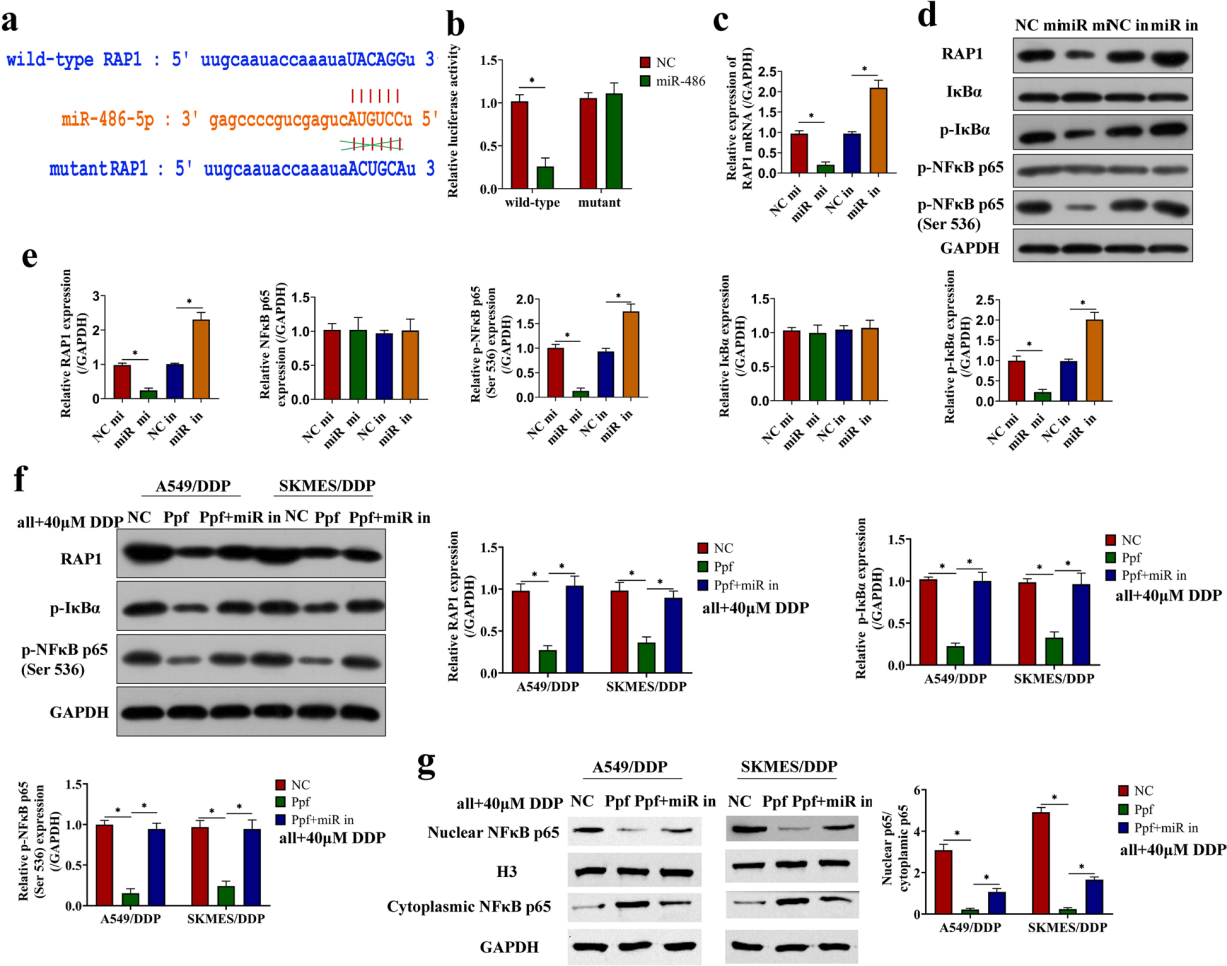
Propofol upregulated miR-486-5p to inactivate the RAP1-NF-κB pathway in NSCLC. **a** The potential target sites between miR-486-5p and 3’UTR of the RAP1 gene was predicted on the online starbase website. **b** The wild-type RAP1 and mutant RAP1 were separately transfected to HEK-293 cells along with NC mimic or miR-486 mimic, and then the targeting relationship between miR-486-5p and RAP1 gene was verified by dual luciferase reporter assay. qRT-PCR(c) and western blot (**d**-**e**) were performed to detect the expression of the RAP1/NF-κB axis in cells with miR-486-5p overexpression or knockdown. **f** The expression of the RAP1/NF-κB axis in DDP-resistant cells treated with miR-486-5p inhibitor, propofol and DDP. **g** The expression of nuclear NF-κB p65 and cytoplasmic NF-κB p65 in DDP-resistant cells treated with miR-486-5p inhibitor, propofol and DDP. *P < 0.05. NC mi, negative control mimic. miR mi, miR-486-5p mimic. NC in, negative control inhibitor. miR in, miR-486-5p inhibitor. NC, control. Ppf, high concentration of propofol. miR in, miR-486-5p inhibitor

As a post-transcriptional regulatory factor, miRNA usually targets functional genes to achieve its functions. Therefore, we continued to study the downstream signaling pathways of miR-486-5p. Through the online starbase website (https://starbase.sysu.edu.cn/agoClipRNA.php?source=mRNA), we predicted the potent binding sites between miR-486-5p and the 3’UTR of the RAP1 gene (Fig. 5a). As previously described, RAP1 was proved to be a key risk factor in the metastasis and DDP resistance of NSCLC [26, 34]. Consequently, we designed a mutant RAP1 sequence around the binding site, and conducted a dual-luciferase reporter gene system assay to verify the targeting relationship. The luciferase activity was reduced by miR-486-5p mimic in HEK-293 cells co-transfected with wild-type RAP1 sequence, while there was no significant difference between miR-486-5p mimic transfection and NC mimic transfection in cells transfected with mutant RAP1 sequence (Fig. 5b). In addition, HEK-293 cells were separately transfected with miR-486-5p mimic or miR-486-5p inhibitor. The expression of RAP1 and the NF-κB axis (p-I**κ**Bα and p-NF-κB p65) were inhibited by miR-486-5p mimic, but promoted by miR-486-5p inhibitor (Fig. 5c-e), which was in consistent with the existed information that RAP1 activates the NF-κB axis to participate in the regulation of cancer progression [25, 35, 36]. In NSCLC cells, propofol inactivated the RAP1/NF-κB axis, which were re-activated by silencing miR-486-5p (Fig. 5f, Figure S5a-d). Consistently, propofol inhibited the translocation of nuclear NF-κB p65 to nucleus in NSCLC cells (Fig. 5g, Figure S5e-f). Those data suggested that propofol upregulated miR-486-5p to inactivate the tumor-promoting RAP1-NF-κB pathway in NSCLC cells.

### Propofol elevated miR-486-5p to enhance DDP-sensitivity in DDP-resistant NSCLC cells via the RAP1-NF-κB axis

**Fig. 6:**
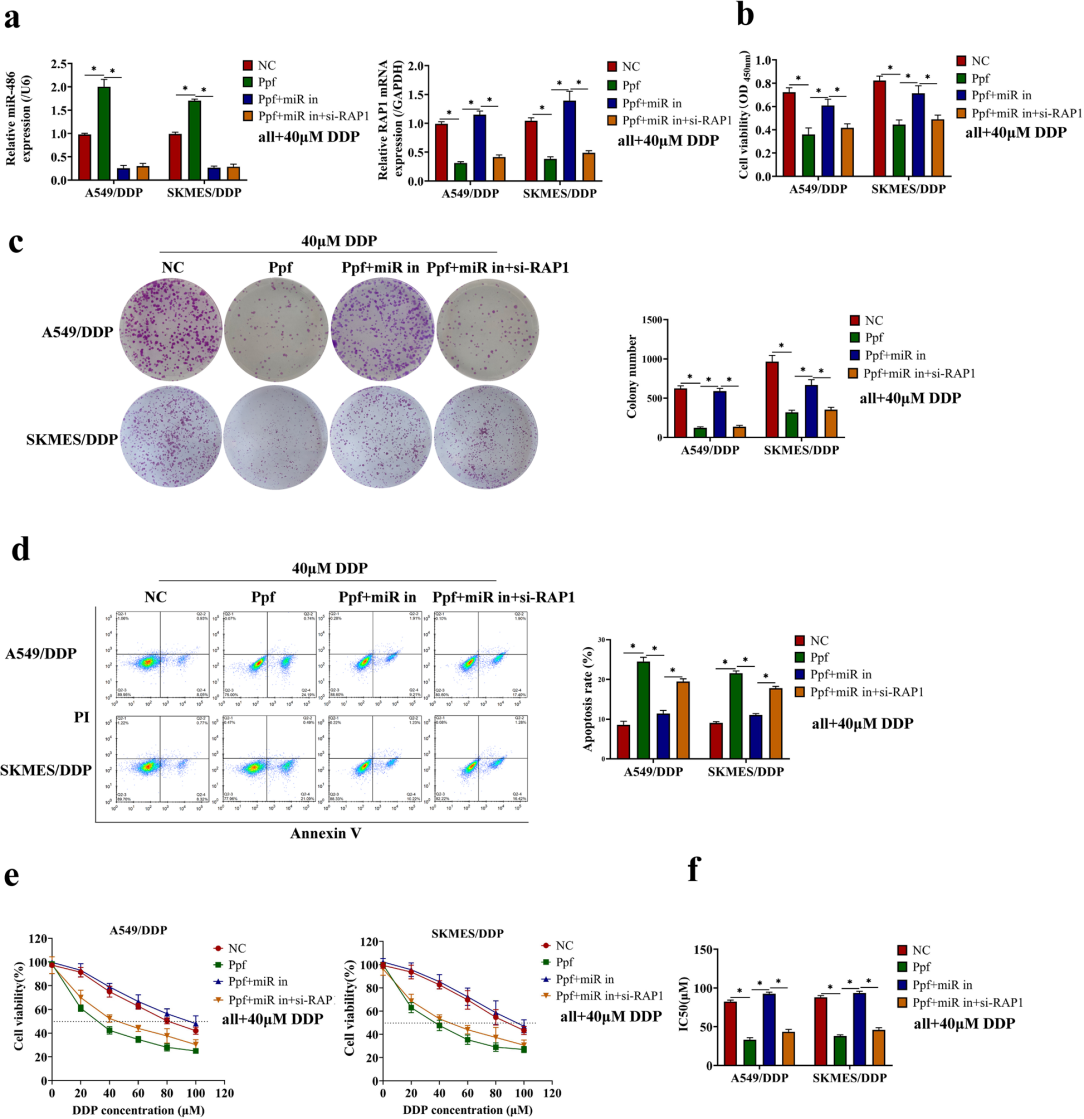
Propofol elevated miR-486-5p to enhance DDP-sensitivity in DDP-resistant NSCLC cells via RAP1-NF-κB axis. The A549/DDP and SKMES/DDP cells were intervened with miR-486-5p inhibitor and siRNA-RAP1, and intervened with 10 μg/mL of propofol and 40 μM of DDP for 24 h. **a** The transfection efficiency was tested by qRT-PCR. **b** Cell viability at 48 h was evaluated through CCK8 assay. **c** The colony formation ability was measured by Giemsa staining. **d** The apoptosis was detected by flow cytometry. **e** The A549/DDP and SKMES/DDP cells were treated with different concentrations of DDP (0, 20, 40, 60, 80, 100 μM), and then cell viability was detected by CCK-8 assay. **f** The IC50 value was calculated according to the dose survival curve. *P < 0.05. NC, negative control. Ppf, high concentration of propofol. miR in, miR-486-5p inhibitor. si-RAP1, siRNA-RAP1

Since we had verified that propofol was capable of regulating DDP-resistance and the cancer-associated miR-486-5p/RAP1-NF-κB axis, so we next explored whether propofol enhanced DDP-sensitivity in NSCLC cells through modulating this signaling pathway. To investigate this issue, we used siRNA-RAP1 to knockdown RAP1 in DDP-resistant and DDP-sensitive NSCLC cells with miR-486-5p ablation (Fig. 6a, Figure S6a), and the cells were further subjected to propofol (10 μg/mL) and DDP co-treatment for 24 h. Results showed that knockdown of RAP1 reversed the effect of miR-486-5p inhibitor on cell viability and apoptosis in the DDP-resistant NSCLC cells co-treated with propofol and DDP (Fig. 6b-d). The similar effect of RAP1 silence on cell viability and apoptosis-related genes were observed in the DDP-sensitive NSCLC cells co-treated by propofol and DDP (Figure S6b-c). At the same time, cell viability at different concentrations of DDP treatment was determined by CCK-8 assay, and results showed that silence of RAP1 enhanced the sensitivity to DDP and reduced the IC50 value in the miR-486-5p-deficient NSCLC cells treated with propofol (Fig. 6e-f, Figure S6d-e). In summary, propofol elevated miR-486-5p to enhance DDP-sensitivity in NSCLC cells via inactivating the RAP1-NF-κB axis.

## Discussion

At present, DDP-based chemotherapy is one of the most effective treatments for NSCLC [37]. Unfortunately, with the progress of chemotherapy, tumors develop drug resistance, and the therapeutic effect of DDP significantly decreases [7]. The mechanism of DDP resistance has been explored for decades, but it has not been fully clarified. Propofol is an intravenous anesthetic frequently used in tumor resection, and has been proved to have antitumor effects in several kinds of cancers [38–41]. In NSCLC, propofol has been reported to induce apoptosis, inhibit cell viability and invasion [27, 42, 43], and it shows good potential in improving drug resistance. In this study, we focused on the DDP resistance of NSCLC, and we used the DDP-sensitive NSCLC cells and DDP-resistant NSCLC cells to test the effects of propofol on DDP resistance. Our data demonstrated that propofol cooperated with DDP to promote cell apoptosis, suppress cell growth, and increase DDP-sensitivity *in vivo* and *in vitro*.

In recent years, the roles of miRNAs in the drug resistance of tumor draw a lot of attention. More and more miRNAs have been sought as the regulators of drug resistance. For example, miR-219a-5p, miR‑139‑5p, miR-186-5p, miR-193a and miR-486-5p are identified to improve the chemotherapy sensitivity in NSCLC [16, 44–47], while miR-126-5p, miR-221 and miR-25-3p are proved to increase the chemotherapy resistance in NSCLC [48–50]. Furthermore, increasing evidence suggests that propofol might affect the biological behavior of cancer cells by regulating the expression of miRNA. A study by Li Y et al. [39] suggests propofol inhibits cell proliferation and metastasis of colorectal cancer cells via upregulating miR-124-3p; Another study by Zheng X et al. [27] indicates propofol inhibits NSCLC cell growth and accelerates apoptosis by downregulating miR-21 expression. In the present study, we found that propofol elevated the miR-486-5p level to increase DDP sensitivity in NSCLC. Previous studies have shown that miR-486-5p is downregulated in NSCLC and regarded as a tumor-inhibitor in lung cancer [13–15]. We found that miR-486-5p levels was decreased during the development of DDP resistance, and propofol could elevate miR-486-5p level in NSCLC cells by promoting the maturation of pri-miR-486 in a m6A-dependent manner. Our findings highlighted the important role of miR-486-5p in the DDP resistance of NSCLC and revealed a novel regulatory mechanism of propofol on the expression of miR-486-5p.

MiRNAs usually regulate the biology behavior by targeting the 3’-untranslated region of mRNA. In this study, the RAP1-NF-κB axis was identified as the downstream target of miR-486-5p, and its expression was significantly affected by propofol. RAP1 is a small GTPase which regulates basic cellular functions through interacting with other proteins [51]. As previous reported, RAP1 is recognized as an oncogene in NSCLC [52], and mediated the DDP resistance by activating the NF-κB pathway [26]. NF-κB pathway is directly associated with chemo-resistance in a variety of cancers, such as breast cancer, pancreatic cancer and colorectal cancer, etc. [53–55]. It is well-known that the excessive activation of NF-κB pathway causes inflammation, oxidative stress reaction and even cell death in normal cells [56–58]. Nevertheless, in tumor cells, the inflammation and oxidative reaction will promote angiogenesis and resistance to apoptosis, leading to subsequent chemo-resistance [59]. All these evidences support the possibility that the RAP1-NF-κB axis become the regulatory signal pathway of propofol in NSCLC. Finally, we demonstrated that propofol elevated miR-486-5p to enhance DDP-resistance via the RAP1-NF-κB axis in NSCLC cells, and the signaling cascade is a novel axis first reported to link with drug resistance of NSCLC.

## Conclusion

In a word, propofol upregulates miR-486-5p to inactivate the RAP1-NF-κB axis in a m6A-dependent manner, thereby increasing DDP-sensitivity in NSCLC. Our study uncovers the molecular mechanism of propofol affecting DDP resistance of NSCLC, which provides a theoretical basis for the application of propofol in the management of chemotherapy resistance in lung cancer.

## Supplementary Information

**Additional file 1. Figure S1.** Propofol enhanced DDP toxicity to DDP-sensitive NSCLC cells. A549 and SKMES cells were pretreated by low concentration of propofol (5μg/mL) or high concentration of propofol (10μg/mL), and then treated with DDP (20μM). (a) The cell viability at different time (0, 24, 48, 72, 96h) was evaluated through CCK8 assay. (b) The apoptosis-related genes, including Bax, Bcl-2 and Caspase-3, were measured by qRT-PCR. *P＜0.05. N, without propofol. L, low concentration of propofol. H, high concentration of propofol.

**Additional file 2. Figure S2. **Propofol increased DDP toxicity to xenografts tumors *in vivo.* A549 cells were injected subcutaneously to nude mice. 7 days after injection of cells, mice were injected intraperitoneally with propofol (35mg/kg) and DDP (5mg/kg) for 3 weeks. (a) The tumor volume was measured every 3 days. (b) On Day 28, mice were sacrificed and the xenografts were removed and weighed. (c) The xenografts tissue was subjected to TUNEL staining and the TUNEL positive rate was regarded as apoptosis rate, and the scale bar = 100mm. (d) The apoptosis-related genes, including Bax, Bcl-2 and Caspase-3, were measured by qRT-PCR. *P＜0.05. NC, negative control.

**Additional file 3. Figure S3. **MiR-486-5p deletion abolished the effect of propofol on DDP-sensitive NSCLC cells. (a) The expression of miR-486-5p in DDP-sensitive cells treated with different concentrations of propofol was determined by qRT-PCR. (b) The expression of miR-486-5p in xenografts tumor established by A549 cells. (c) The A549 and SKMES cells were transfected with or without miR-486-5p inhibitor, and intervened with high concentration of propofol (10μg/mL), and the effect on miR-486-5p expression was verified by qRT-PCR. (d) Cell viability was measured by CCK-8 assay. (e) The apoptosis-related genes, including Bax, Bcl-2 and Caspase-3, were measured by qRT-PCR. *P＜0.05. N, without propofol. L, low concentration of propofol. H, high concentration of propofol. NC, negative control. Ppf, high concentration of propofol. miR in, miR-486-5p inhibitor.

**Additional file 4. Figure S4. **Propofol promoted the maturation of pri-miR-486-5p in NSCLC cells in a m6A-dependent manner. (a-b) The expression of pri-miR-486 and miR-486 in A549, A549/DDP, SKMES and SKMES/DDP. (c-d) The expression of pri-miR-486 in DDP-resistant cells or DDP-sensitive cells co-treated with DDP (20μM), low concentration of propofol (5μg/mL) or high concentration of propofol (10μg/mL). (e-f）The xenografts tumor model mice established by A549/DDP, SKMES/DDP or A549 cells were injected intraperitoneally with propofol (35mg/kg) and DDP (5mg/kg) for 3 weeks, and the expression of pri-miR-486 in the xenografts tumor tissues was detected by qRT-PCR. (g) The enrichment of m6A in pri-miR-486 was evaluated by RNA immunoprecipitation assay in NSCLC cells treated with or without 10μg/mL propofol. (h-i) The expression of pri-miR-486 (h) and miR-486 (i) in A549, SKMES, A549/DDP and SKMES/DDP cells co-treated with 10μg/mL propofol and siRNA-METTL3. *P＜0.05. NC, negative control. Ppf, high concentration of propofol. miR in, miR-486-5p inhibitor. KD, knockdown.

**Additional file 5. Figure S5. **Propofol upregulated miR-486-5p to inactivate the RAP1-NF-κB pathway in DDP-sensitive NSCLC cells. (a-d) The expression of the RAP1/NF-κB axis in DDP-resistant cells treated with miR-486-5p inhibitor, propofol and DDP. (e-f) The expression of nuclear NF-κB p65 and cytoplasmic NF-κB p65 in DDP-resistant cells treated with miR-486-5p inhibitor, propofol and DDP. *P＜0.05. NC, negative control. Ppf, high concentration of propofol. miR in, miR-486-5p inhibitor.

**Additional file 6. Figure S6. **Propofol elevated miR-486-5p to enhance DDP toxicity in DDP-sensitive NSCLC cells via RAP1-NF-κB axis. The A549 and SKMES cells were intervened with miR-486-5p inhibitor and siRNA-RAP1, and intervened with 10μg/mL propofol and 20μM of DDP for 24h. (a) The transfection efficiency was tested by qRT-PCR. (b) Cell viability at 48h was evaluated through CCK8 assay. (c) The apoptosis-related genes, including Bax, Bcl-2 and Caspase-3, were measured by qRT-PCR. (d) The cells were treated with different concentrations of DDP (0, 10, 20, 30, 40, 50mM), and then cell viability was detected by CCK-8 assay. (e) The IC50 value was calculated according to the dose survival curve. *P＜0.05. NC, negative control. Ppf, high concentration of propofol. miR in, miR-486-5p inhibitor. si-RAP1, siRNA-RAP1.

**Additional file 7. **

## Data Availability

The data generated during the study are included within the manuscript file.

## References

[1] Bray F, Ferlay J, Soerjomataram I, Siegel RL, Torre LA, Jemal A (2018). Global cancer statistics 2018: GLOBOCAN estimates of incidence and mortality worldwide for 36 cancers in 185 countries. CA Cancer J Clin.

[2] Pan X, Chen Y, Shen Y, Tantai J (2019). Knockdown of TRIM65 inhibits autophagy and cisplatin resistance in A549/DDP cells by regulating miR-138-5p/ATG7. Cell Death Dis.

[3] Chen B, Shen Z, Wu D, Xie X, Xu X, Lv L (2019). Glutathione peroxidase 1 promotes NSCLC resistance to cisplatin via ROS-induced activation of PI3K/AKT pathway. Biomed Res Int.

[4] Cruz-Bermúdez A, Laza-Briviesca R, Vicente-Blanco RJ, García-Grande A, Coronado MJ, Laine-Menéndez S (2019). Cisplatin resistance involves a metabolic reprogramming through ROS and PGC-1α in NSCLC which can be overcome by OXPHOS inhibition. Free Radic Biol Med.

[5] Wang W, Zhao M, Cui L, Ren Y, Zhang J, Chen J (2020). Characterization of a novel HDAC/RXR/HtrA1 signaling axis as a novel target to overcome cisplatin resistance in human non-small cell lung cancer. Mol Cancer.

[6] Qian J, Shen S, Chen W, Chen N (2018). Propofol reversed hypoxia-induced docetaxel resistance in prostate cancer cells by preventing epithelial-mesenchymal transition by inhibiting hypoxia-inducible factor 1α. Biomed Res Int.

[7] Sun Y, Peng YB, Ye LL, Ma LX, Zou MY, Cheng ZG (2020). Propofol inhibits proliferation and cisplatin resistance in ovarian cancer cells through regulating the microRNA-374a/forkhead box O1 signaling axis. Mol Med Rep.

[8] Liu WZ, Liu N (2018). Propofol inhibits lung cancer A549 cell growth and epithelial-mesenchymal transition process by upregulation of MicroRNA-1284. Oncol Res.

[9] Huang Y, Lei L, Liu Y (2020). Propofol improves sensitivity of lung cancer cells to cisplatin and its mechanism. Med Sci Monit.

[10] Lang B, Zhao S (2018). miR-486 functions as a tumor suppressor in esophageal cancer by targeting CDK4/BCAS2. Oncol Rep.

[11] Li H, Mou Q, Li P, Yang Z, Wang Z, Niu J (2019). MiR-486-5p inhibits IL-22-induced epithelial-mesenchymal transition of breast cancer cell by repressing Dock1. J Cancer.

[12] Zhang Y, Fu J, Zhang Z, Qin H (2018). miR-486-5p regulates the migration and invasion of colorectal cancer cells through targeting PIK3R1. Oncol Lett.

[13] Tian F, Wang J, Ouyang T, Lu N, Lu J, Shen Y (2019). MiR-486-5p serves as a good biomarker in nonsmall cell lung cancer and suppresses cell growth with the involvement of a target PIK3R1. Front Genet.

[14] Wang A, Zhu J, Li J, Du W, Zhang Y, Cai T (2020). Downregulation of KIAA1199 by miR-486-5p suppresses tumorigenesis in lung cancer. Cancer Med.

[15] Yu S, Geng S, Hu Y (2018). miR-486-5p inhibits cell proliferation and invasion through repressing GAB2 in non-small cell lung cancer. Oncol Lett.

[16] Jin X, Pang W, Zhang Q, Huang H (2019). MicroRNA-486-5p improves nonsmall-cell lung cancer chemotherapy sensitivity and inhibits epithelial-mesenchymal transition by targeting twinfilin actin binding protein 1. J Int Med Res.

[17] Okamura S, Yoshino H, Kuroshima K, Tsuruda M, Osako Y, Sakaguchi T (2021). EHHADH contributes to cisplatin resistance through regulation by tumor-suppressive microRNAs in bladder cancer. BMC Cancer.

[18] Pinelli S, Alinovi R, Poli D, Corradi M, Pelosi G, Tiseo M (2021). Overexpression of microRNA-486 affects the proliferation and chemosensitivity of mesothelioma cell lines by targeting PIM1. Int J Mol Med.

[19] Salimian J, Baradaran B, AzimzadehJamalkandi S, Moridikia A, Ahmadi A (2020). MiR-486-5p enhances cisplatin sensitivity of human muscle-invasive bladder cancer cells by induction of apoptosis and down-regulation of metastatic genes. Urol Oncol.

[20] Yang N, Liang Y, Yang P, Yang T, Jiang L (2017). Propofol inhibits lung cancer cell viability and induces cell apoptosis by upregulating microRNA-486 expression. Braz J Med Biol Res.

[21] Looi CK, Hii LW, Ngai SC, Leong CO, Mai CW (2020). The role of ras-associated protein 1 (Rap1) in cancer: bad actor or good player?. Biomedicines.

[22] Moon EY, Pyo S (2007). Lipopolysaccharide stimulates Epac1-mediated Rap1/NF-kappaB pathway in Raw 264.7 murine macrophages. Immunol Lett..

[23] Zhang Y, Chiu S, Liang X, Gao F, Zhang Z, Liao S (2015). Rap1-mediated nuclear factor-kappaB (NF-κB) activity regulates the paracrine capacity of mesenchymal stem cells in heart repair following infarction. Cell Death Discov.

[24] Ryan SL, Beard S, Barr MP, Umezawa K, Heavey S, Godwin P (2019). Targeting NF-κB-mediated inflammatory pathways in cisplatin-resistant NSCLC. Lung Cancer.

[25] Teo H, Ghosh S, Luesch H, Ghosh A, Wong ET, Malik N (2010). Telomere-independent Rap1 is an IKK adaptor and regulates NF-kappaB-dependent gene expression. Nat Cell Biol.

[26] Xiao L, Lan X, Shi X, Zhao K, Wang D, Wang X (2017). Cytoplasmic RAP1 mediates cisplatin resistance of non-small cell lung cancer. Cell Death Dis.

[27] Zheng X, Dong L, Zhao S, Li Q, Liu D, Zhu X (2020). Propofol affects non-small-cell lung cancer cell biology by regulating the miR-21/PTEN/AKT pathway in vitro and in vivo. Anesth Analg.

[28] Fu D, Lu C, Qu X, Li P, Chen K, Shan L (2019). LncRNA TTN-AS1 regulates osteosarcoma cell apoptosis and drug resistance via the miR-134-5p/MBTD1 axis. Aging (Albany NY).

[29] Gao ZJ, Yuan WD, Yuan JQ, Yuan K, Wang Y (2018). miR-486-5p functions as an oncogene by targeting PTEN in non-small cell lung cancer. Pathol Res Pract.

[30] Tian F, Shen Y, Chen Z, Li R, Lu J, Ge Q (2016). Aberrant miR-181b-5p and miR-486-5p expression in serum and tissue of non-small cell lung cancer. Gene.

[31] Xing Z, Zhang Z, Gao Y, Zhang X, Kong X, Zhang J (2020). The lncRNA LINC01194/miR-486-5p Axis facilitates malignancy in non-small cell lung cancer via regulating CDK4. Onco Targets Ther.

[32] Wang P, Wang Z, Zhang M, Wu Q, Shi F, Yuan S (2021). KIAA1429 and ALKBH5 oppositely influence aortic dissection progression via regulating the maturation of Pri-miR-143-3p in an m6A-dependent manner. Front Cell Dev Biol.

[33] Zhang S, Zhao S, Qi Y, Li B, Wang H, Pan Z (2022). SPI1-induced downregulation of FTO promotes GBM progression by regulating pri-miR-10a processing in an m6A-dependent manner. Mol Ther Nucleic Acids.

[34] Kan J, Fu B, Zhou R, Zhou D, Huang Y, Zhao H (2022). He-Chan Pian inhibits the metastasis of non-small cell lung cancer via the miR-205-5p-mediated regulation of the GREM1/Rap1 signaling pathway. Phytomedicine.

[35] Lian S, Meng L, Liu C, Xing X, Song Q, Dong B (2013). PRL-3 activates NF-κB signaling pathway by interacting with RAP1. Biochem Biophys Res Commun.

[36] Mo SJ, Hou X, Hao XY, Cai JP, Liu X, Chen W (2018). EYA4 inhibits hepatocellular carcinoma growth and invasion by suppressing NF-κB-dependent RAP1 transactivation. Cancer Commun (Lond).

[37] Rossi A, Di Maio M (2016). Platinum-based chemotherapy in advanced non-small-cell lung cancer: optimal number of treatment cycles. Expert Rev Anticancer Ther.

[38] Gao Y, Yu X, Zhang F, Dai J (2019). Propofol inhibits pancreatic cancer progress under hypoxia via ADAM8. J Hepatobiliary Pancreat Sci.

[39] Li Y, Dong W, Yang H, Xiao G (2020). Propofol suppresses proliferation and metastasis of colorectal cancer cells by regulating miR-124–3p.1/AKT3. Biotechnol Lett.

[40] Ni YJ, Lu J, Zhou HM (2019). Propofol suppresses proliferation, migration and invasion of gastric cancer cells via regulating miR-29/MMP-2 axis. Eur Rev Med Pharmacol Sci.

[41] Tian D, Tian M, Ma ZM, Zhang LL, Cui YF, Li JL (2020). Anesthetic propofol epigenetically regulates breast cancer trastuzumab resistance through IL-6/miR-149-5p axis. Sci Rep.

[42] Xing SG, Zhang KJ, Qu JH, Ren YD, Luan Q (2018). Propofol induces apoptosis of non-small cell lung cancer cells via ERK1/2-dependent upregulation of PUMA. Eur Rev Med Pharmacol Sci.

[43] Yang N, Liang Y, Yang P, Ji F (2017). Propofol suppresses LPS-induced nuclear accumulation of HIF-1α and tumor aggressiveness in non-small cell lung cancer. Oncol Rep.

[44] Rao C, Miao X, Zhao G, Zhang C, Shen H, Dong C (2019). MiR-219a-5p enhances cisplatin sensitivity of human non-small cell lung cancer by targeting FGF9. Biomed Pharmacother.

[45] Du H, Bao Y, Liu C, Zhong A, Niu Y, Tang X (2021). miR-139-5p enhances cisplatin sensitivity in non-small cell lung cancer cells by inhibiting cell proliferation and promoting apoptosis via the targeting of Homeobox protein Hox-B2. Mol Med Rep.

[46] Liu X, Zhou X, Chen Y, Huang Y, He J, Luo H (2020). miR-186-5p targeting SIX1 inhibits cisplatin resistance in non-small-cell lung cancer cells (NSCLCs). Neoplasma.

[47] Wu H, Mu X, Liu L, Wu H, Hu X, Chen L (2020). Bone marrow mesenchymal stem cells-derived exosomal microRNA-193a reduces cisplatin resistance of non-small cell lung cancer cells via targeting LRRC1. Cell Death Dis.

[48] Liu B, Wang R, Liu H (2021). mir-126-5p promotes cisplatin sensitivity of non-small-cell lung cancer by inhibiting ADAM9. Biomed Res Int.

[49] Guo S, Zhang L, Zhang Y, Wu Z, He D, Li X (2019). Long non-coding RNA TUG1 enhances chemosensitivity in non-small cell lung cancer by impairing microRNA-221-dependent PTEN inhibition. Aging (Albany NY).

[50] Sun B, Hu N, Cong D, Chen K, Li J (2021). MicroRNA-25-3p promotes cisplatin resistance in non-small-cell lung carcinoma (NSCLC) through adjusting PTEN/PI3K/AKT route. Bioengineered.

[51] Zhang YL, Wang RC, Cheng K, Ring BZ, Su L (2017). Roles of Rap1 signaling in tumor cell migration and invasion. Cancer Biol Med.

[52] Lu J, Zhou L, Wu B, Duan Y, Sun Y, Gu L (2020). MiR-501-3p functions as a tumor suppressor in non-small cell lung cancer by downregulating RAP1A. Exp Cell Res.

[53] Kumar S, Nandi A, Singh S, Regulapati R, Li N, Tobias JW (2021). Dll1(+) quiescent tumor stem cells drive chemoresistance in breast cancer through NF-κB survival pathway. Nat Commun.

[54] Meng Q, Liang C, Hua J, Zhang B, Liu J, Zhang Y (2020). A miR-146a-5p/TRAF6/NF-kB p65 axis regulates pancreatic cancer chemoresistance: functional validation and clinical significance. Theranostics.

[55] Wang D, Yang L, Yu W, Wu Q, Lian J, Li F (2019). Colorectal cancer cell-derived CCL20 recruits regulatory T cells to promote chemoresistance via FOXO1/CEBPB/NF-κB signaling. J Immunother Cancer.

[56] Jiang F, Xu XR, Li WM, Xia K, Wang LF, Yang XC (2020). Monotropein alleviates H2O2-induced inflammation, oxidative stress and apoptosis via NF-κB/AP-1 signaling. Mol Med Rep.

[57] Liu H, Wu X, Luo J, Wang X, Guo H, Feng D (2019). Pterostilbene attenuates astrocytic inflammation and neuronal oxidative injury after ischemia-reperfusion by inhibiting NF-κB phosphorylation. Front Immunol.

[58] Zhu MM, Wang L, Yang D, Li C, Pang ST, Li XH (2019). Wedelolactone alleviates doxorubicin-induced inflammation and oxidative stress damage of podocytes by IκK/IκB/NF-κB pathway. Biomed Pharmacother.

[59] Mortezaee K, Najafi M, Farhood B, Ahmadi A, Shabeeb D, Musa AE (2019). NF-κB targeting for overcoming tumor resistance and normal tissues toxicity. J Cell Physiol.

