# Supplementary figures and images for: Anesthetic propofol enhances cisplatin-sensitivity of non-small cell lung cancer cells through N6-methyladenosine-dependently regulating the miR-486-5p/RAP1-NF-κB axis
Source: BMC Cancer. 2022 Jul 14;22:765. doi: 10.1186/s12885-022-09848-y (PMC9281112; doi:10.1186/s12885-022-09848-y)

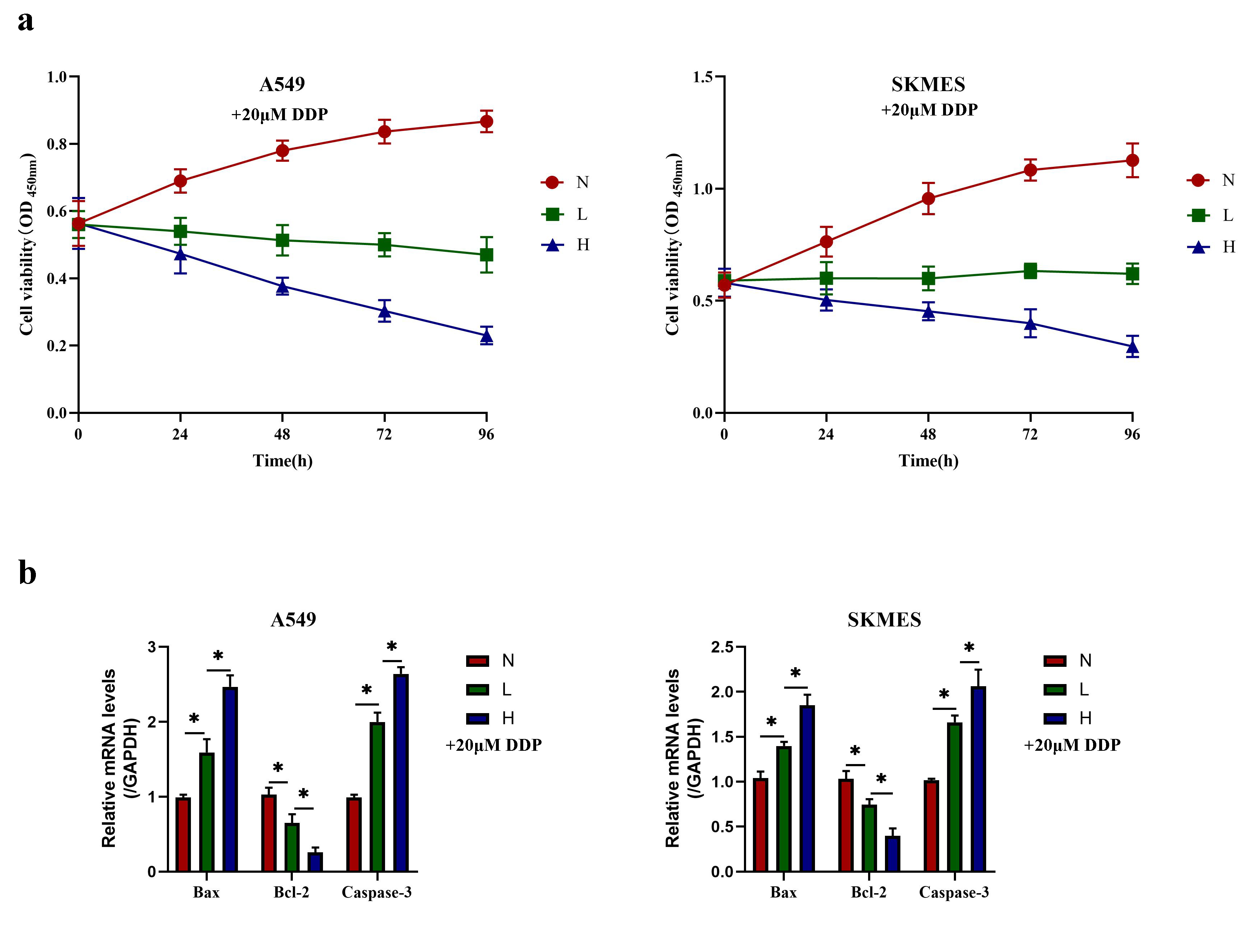

Supplement: Supplementary file 1 — Additional file 1. Figure S1. Propofol enhanced DDP toxicity to DDP-sensitive NSCLC cells. A549 and SKMES cells were pretreated by low concentration of propofol (5μg/mL) or high concentration of propofol (10μg/mL), and then treated with DDP (20μM). (a) The cell viability at different time (0, 24, 48, 72, 96h) was evaluated through CCK8 assay. (b) The apoptosis-related genes, including Bax, Bcl-2 and Caspase-3, were measured by qRT-PCR. *P＜0.05. N, without propofol. L, low concentration of propofol. H, high concentration of propofol. [file 12885_2022_9848_MOESM1_ESM.jpg]

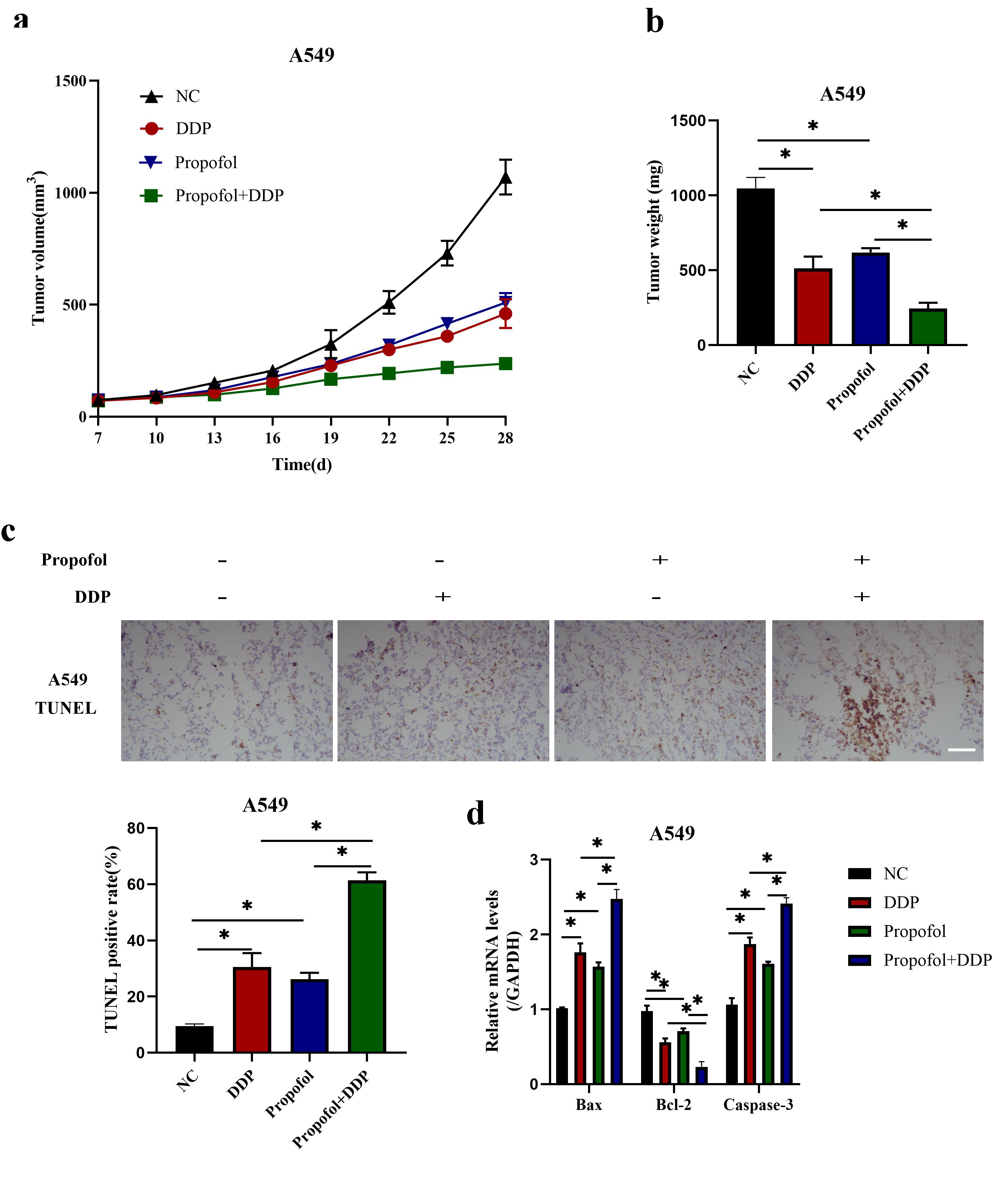

Supplement: Supplementary file 2 — Additional file 2. Figure S2. Propofol increased DDP toxicity to xenografts tumors in vivo. A549 cells were injected subcutaneously to nude mice. 7 days after injection of cells, mice were injected intraperitoneally with propofol (35mg/kg) and DDP (5mg/kg) for 3 weeks. (a) The tumor volume was measured every 3 days. (b) On Day 28, mice were sacrificed and the xenografts were removed and weighed. (c) The xenografts tissue was subjected to TUNEL staining and the TUNEL positive rate was regarded as apoptosis rate, and the scale bar = 100mm. (d) The apoptosis-related genes, including Bax, Bcl-2 and Caspase-3, were measured by qRT-PCR. *P＜0.05. NC, negative control. [file 12885_2022_9848_MOESM2_ESM.jpg]

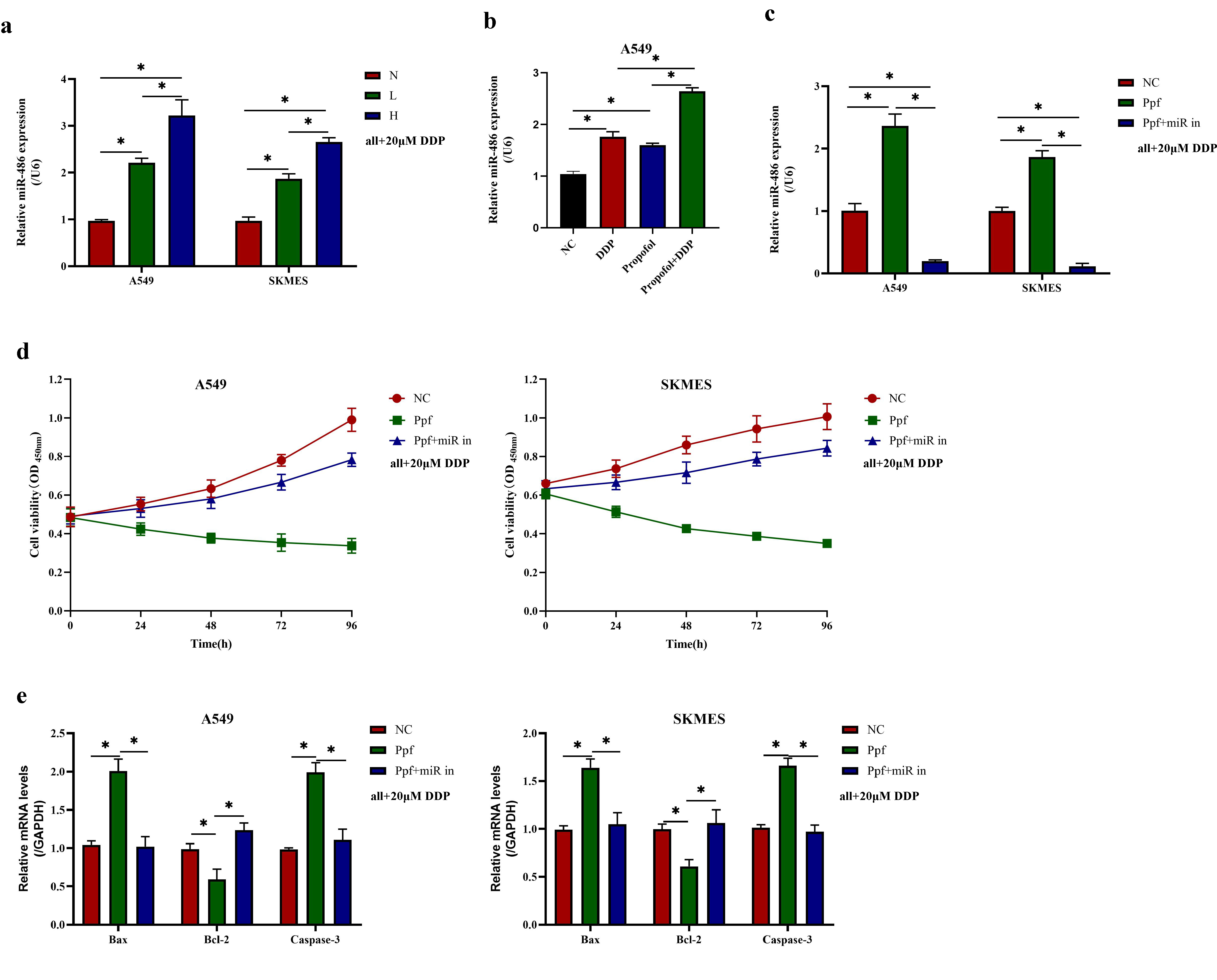

Supplement: Supplementary file 3 — Additional file 3. Figure S3. MiR-486-5p deletion abolished the effect of propofol on DDP-sensitive NSCLC cells. (a) The expression of miR-486-5p in DDP-sensitive cells treated with different concentrations of propofol was determined by qRT-PCR. (b) The expression of miR-486-5p in xenografts tumor established by A549 cells. (c) The A549 and SKMES cells were transfected with or without miR-486-5p inhibitor, and intervened with high concentration of propofol (10μg/mL), and the effect on miR-486-5p expression was verified by qRT-PCR. (d) Cell viability was measured by CCK-8 assay. (e) The apoptosis-related genes, including Bax, Bcl-2 and Caspase-3, were measured by qRT-PCR. *P＜0.05. N, without propofol. L, low concentration of propofol. H, high concentration of propofol. NC, negative control. Ppf, high concentration of propofol. miR in, miR-486-5p inhibitor. [file 12885_2022_9848_MOESM3_ESM.jpg]

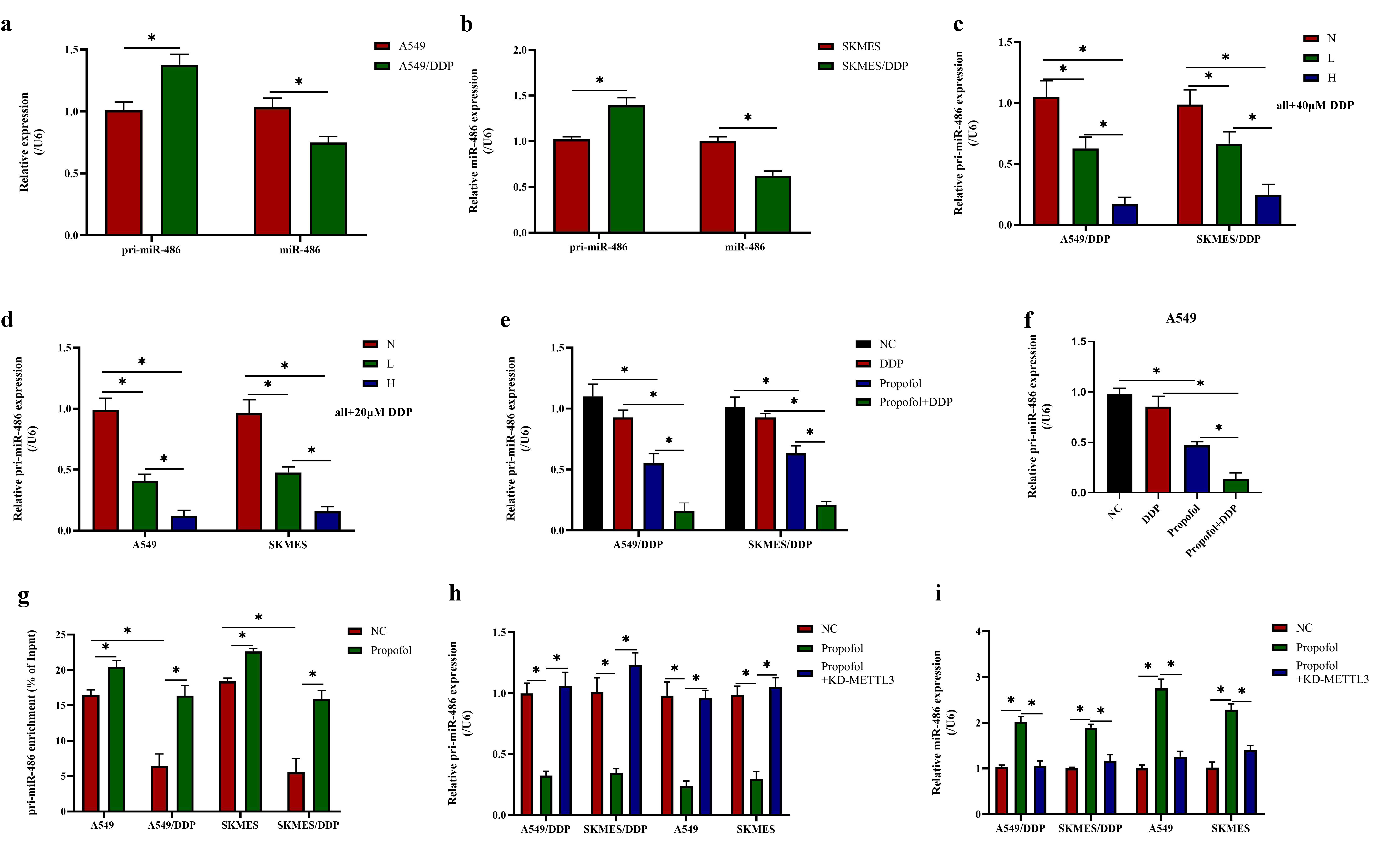

Supplement: Supplementary file 4 — Additional file 4. Figure S4. Propofol promoted the maturation of pri-miR-486-5p in NSCLC cells in a m6A-dependent manner. (a-b) The expression of pri-miR-486 and miR-486 in A549, A549/DDP, SKMES and SKMES/DDP. (c-d) The expression of pri-miR-486 in DDP-resistant cells or DDP-sensitive cells co-treated with DDP (20μM), low concentration of propofol (5μg/mL) or high concentration of propofol (10μg/mL). (e-f）The xenografts tumor model mice established by A549/DDP, SKMES/DDP or A549 cells were injected intraperitoneally with propofol (35mg/kg) and DDP (5mg/kg) for 3 weeks, and the expression of pri-miR-486 in the xenografts tumor tissues was detected by qRT-PCR. (g) The enrichment of m6A in pri-miR-486 was evaluated by RNA immunoprecipitation assay in NSCLC cells treated with or without 10μg/mL propofol. (h-i) The expression of pri-miR-486 (h) and miR-486 (i) in A549, SKMES, A549/DDP and SKMES/DDP cells co-treated with 10μg/mL propofol and siRNA-METTL3. *P＜0.05. NC, negative control. Ppf, high concentration of propofol. miR in, miR-486-5p inhibitor. KD, knockdown. [file 12885_2022_9848_MOESM4_ESM.jpg]

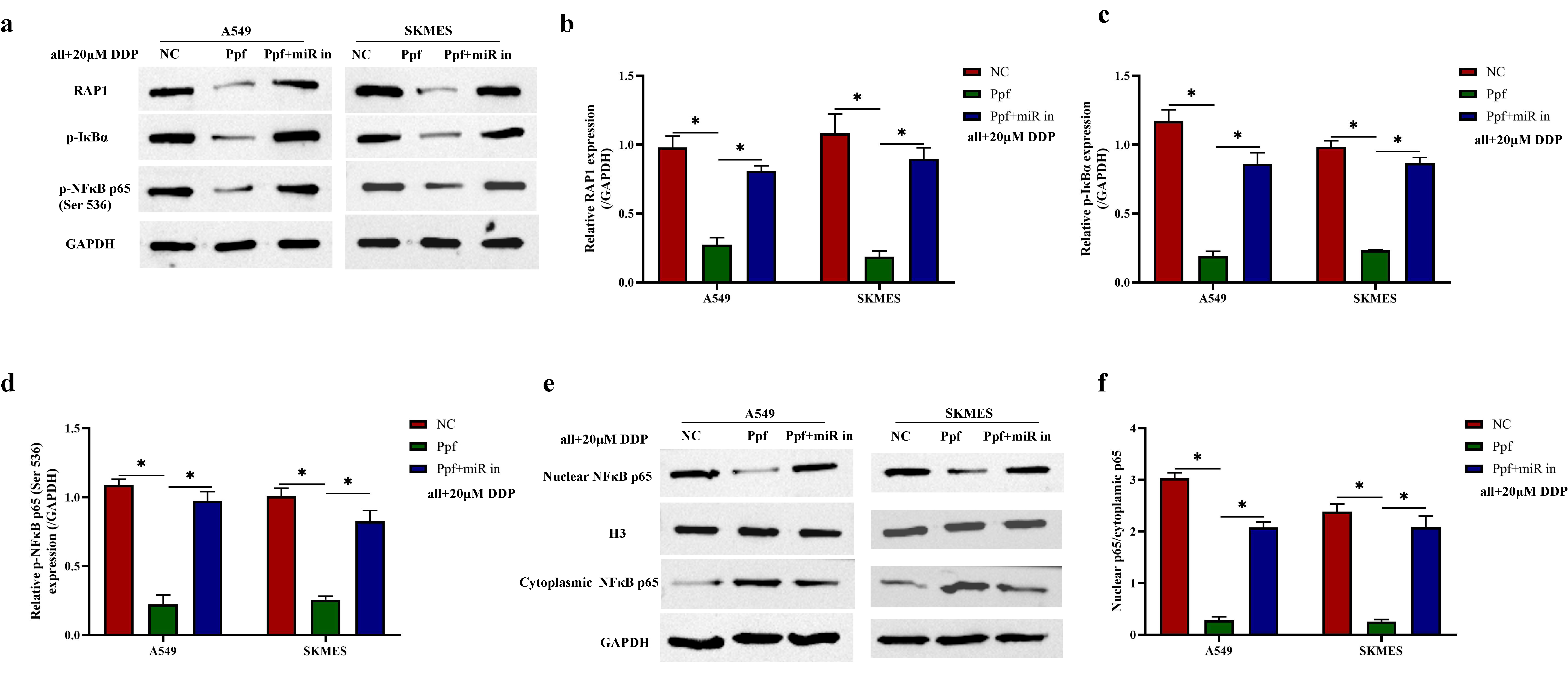

Supplement: Supplementary file 5 — Additional file 5. Figure S5. Propofol upregulated miR-486-5p to inactivate the RAP1-NF-κB pathway in DDP-sensitive NSCLC cells. (a-d) The expression of the RAP1/NF-κB axis in DDP-resistant cells treated with miR-486-5p inhibitor, propofol and DDP. (e-f) The expression of nuclear NF-κB p65 and cytoplasmic NF-κB p65 in DDP-resistant cells treated with miR-486-5p inhibitor, propofol and DDP. *P＜0.05. NC, negative control. Ppf, high concentration of propofol. miR in, miR-486-5p inhibitor. [file 12885_2022_9848_MOESM5_ESM.jpg]

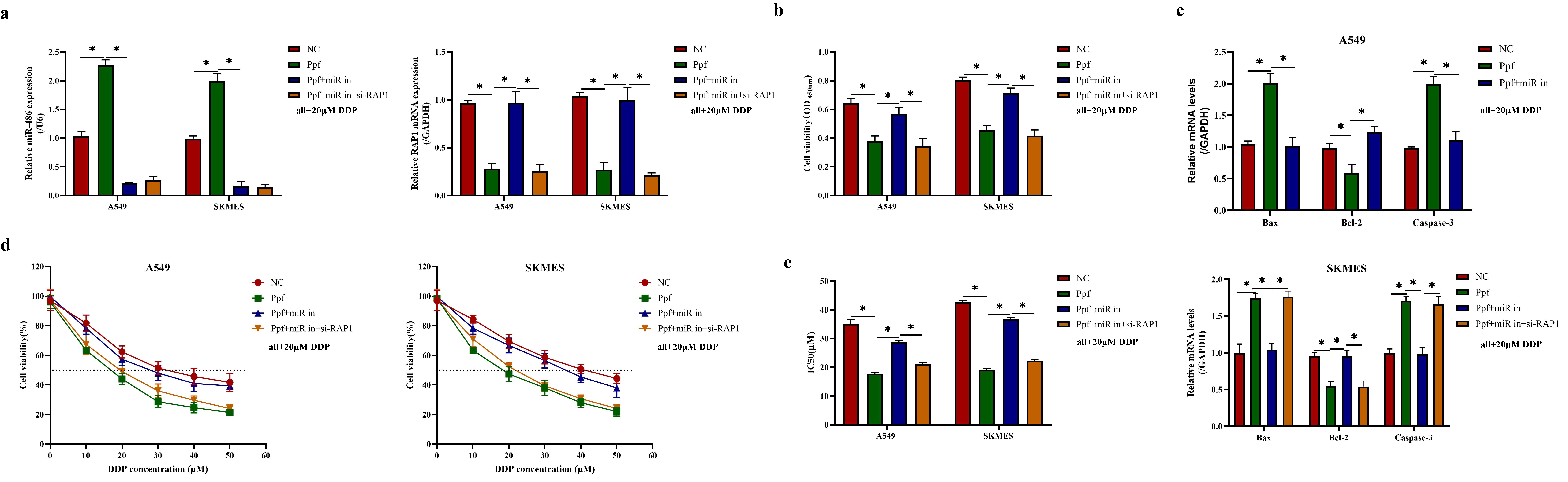

Supplement: Supplementary file 6 — Additional file 6. Figure S6. Propofol elevated miR-486-5p to enhance DDP toxicity in DDP-sensitive NSCLC cells via RAP1-NF-κB axis. The A549 and SKMES cells were intervened with miR-486-5p inhibitor and siRNA-RAP1, and intervened with 10μg/mL propofol and 20μM of DDP for 24h. (a) The transfection efficiency was tested by qRT-PCR. (b) Cell viability at 48h was evaluated through CCK8 assay. (c) The apoptosis-related genes, including Bax, Bcl-2 and Caspase-3, were measured by qRT-PCR. (d) The cells were treated with different concentrations of DDP (0, 10, 20, 30, 40, 50mM), and then cell viability was detected by CCK-8 assay. (e) The IC50 value was calculated according to the dose survival curve. *P＜0.05. NC, negative control. Ppf, high concentration of propofol. miR in, miR-486-5p inhibitor. si-RAP1, siRNA-RAP1. [file 12885_2022_9848_MOESM6_ESM.jpg]
