# Supplementary material for: Anesthetic propofol enhances cisplatin-sensitivity of non-small cell lung cancer cells through N6-methyladenosine-dependently regulating the miR-486-5p/RAP1-NF-κB axis
Source: BMC Cancer. 2022 Jul 14;22:765. doi: 10.1186/s12885-022-09848-y (PMC9281112; doi:10.1186/s12885-022-09848-y)
Supplement: Supplementary file 7 — Additional file 7. [file 12885_2022_9848_MOESM7_ESM.docx]

**Original gels and blots**

**Figure 5d**


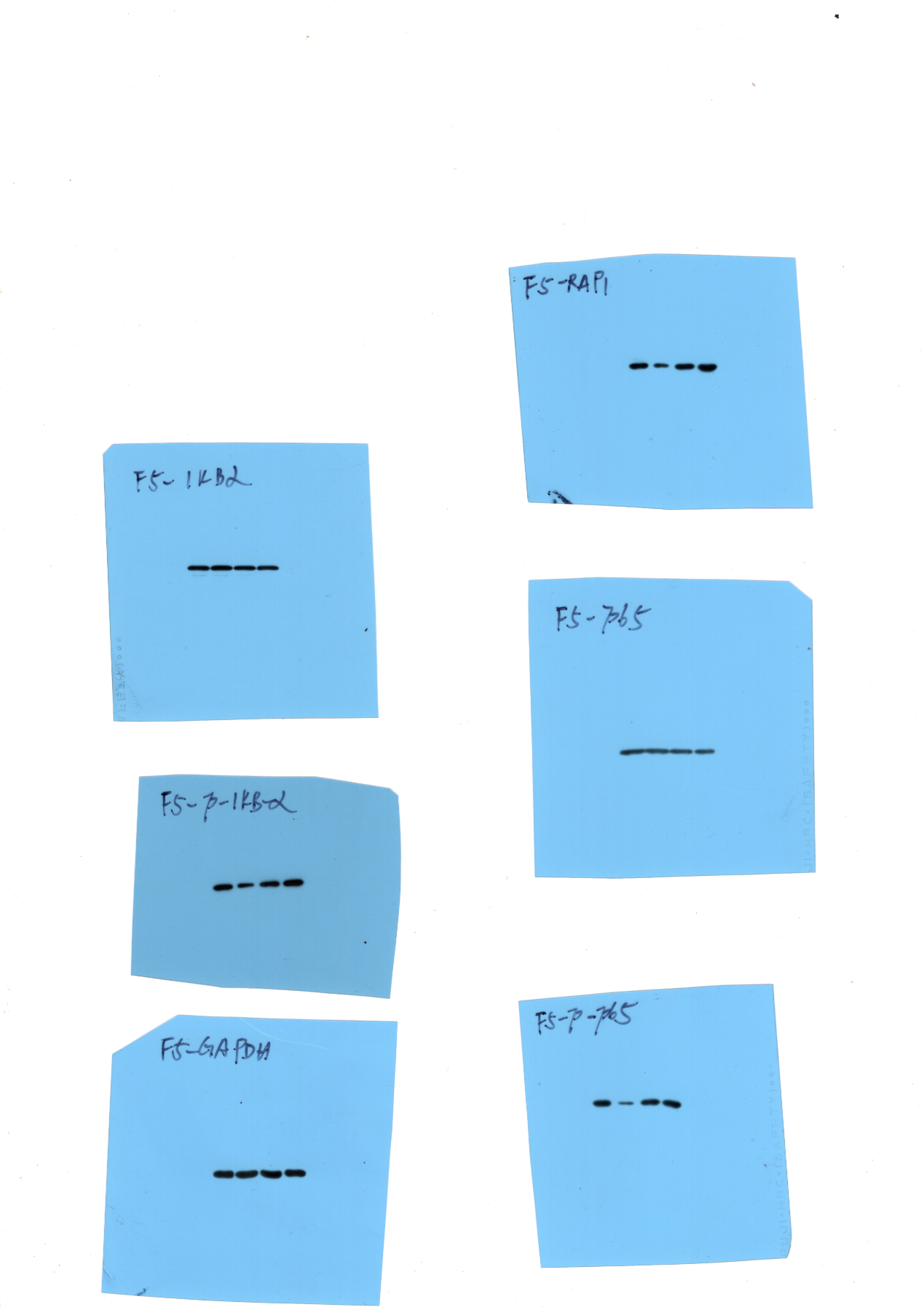

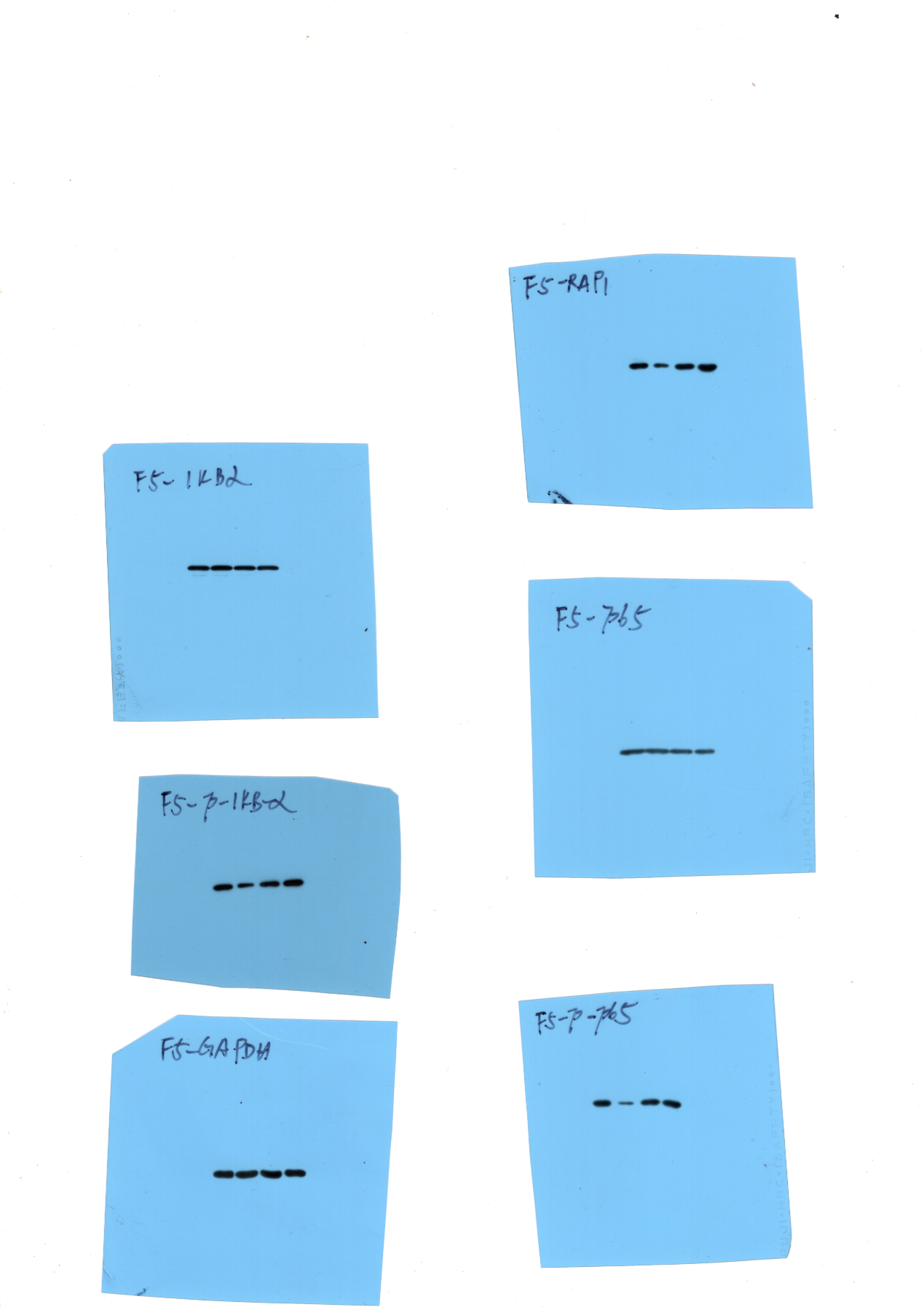

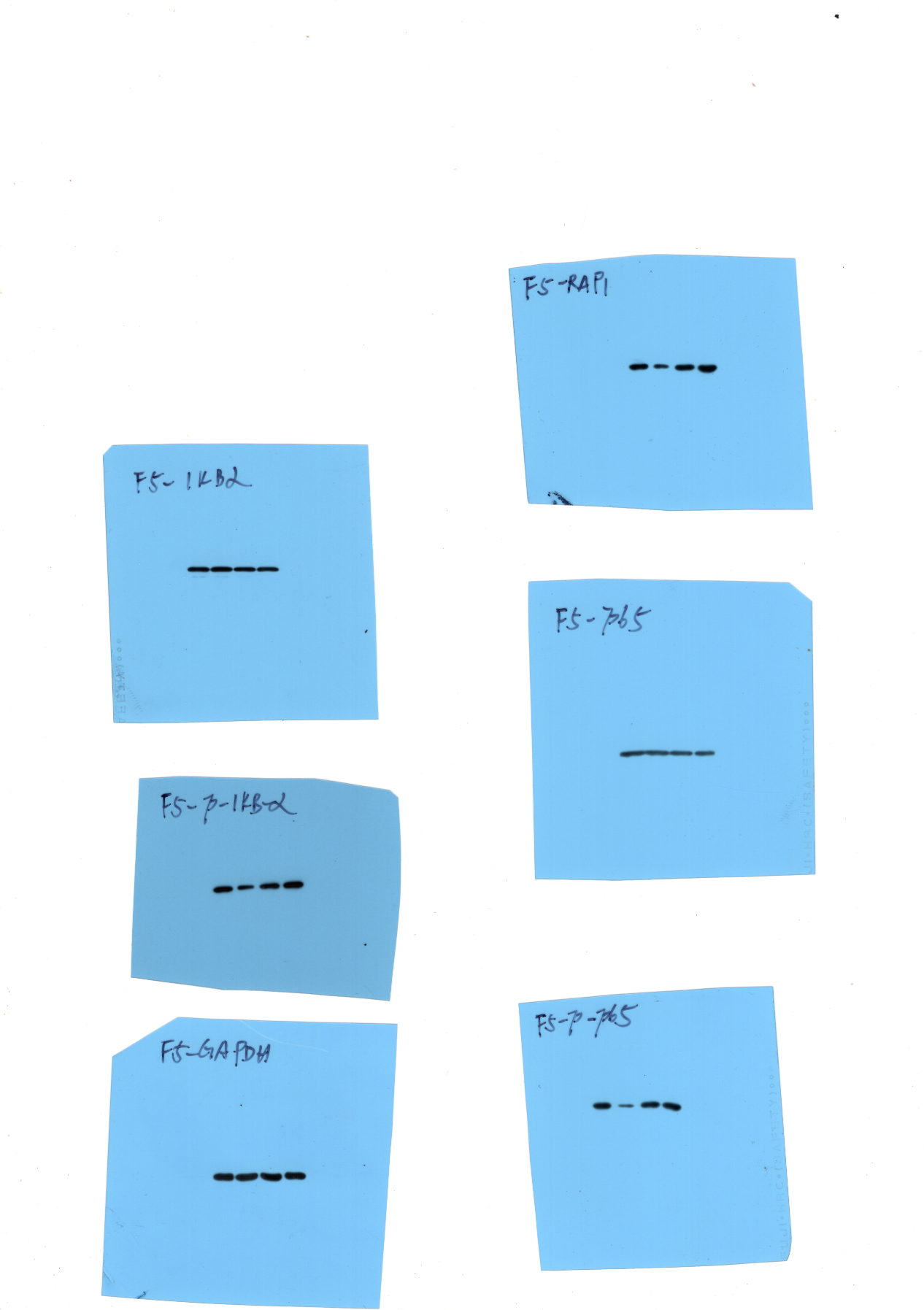


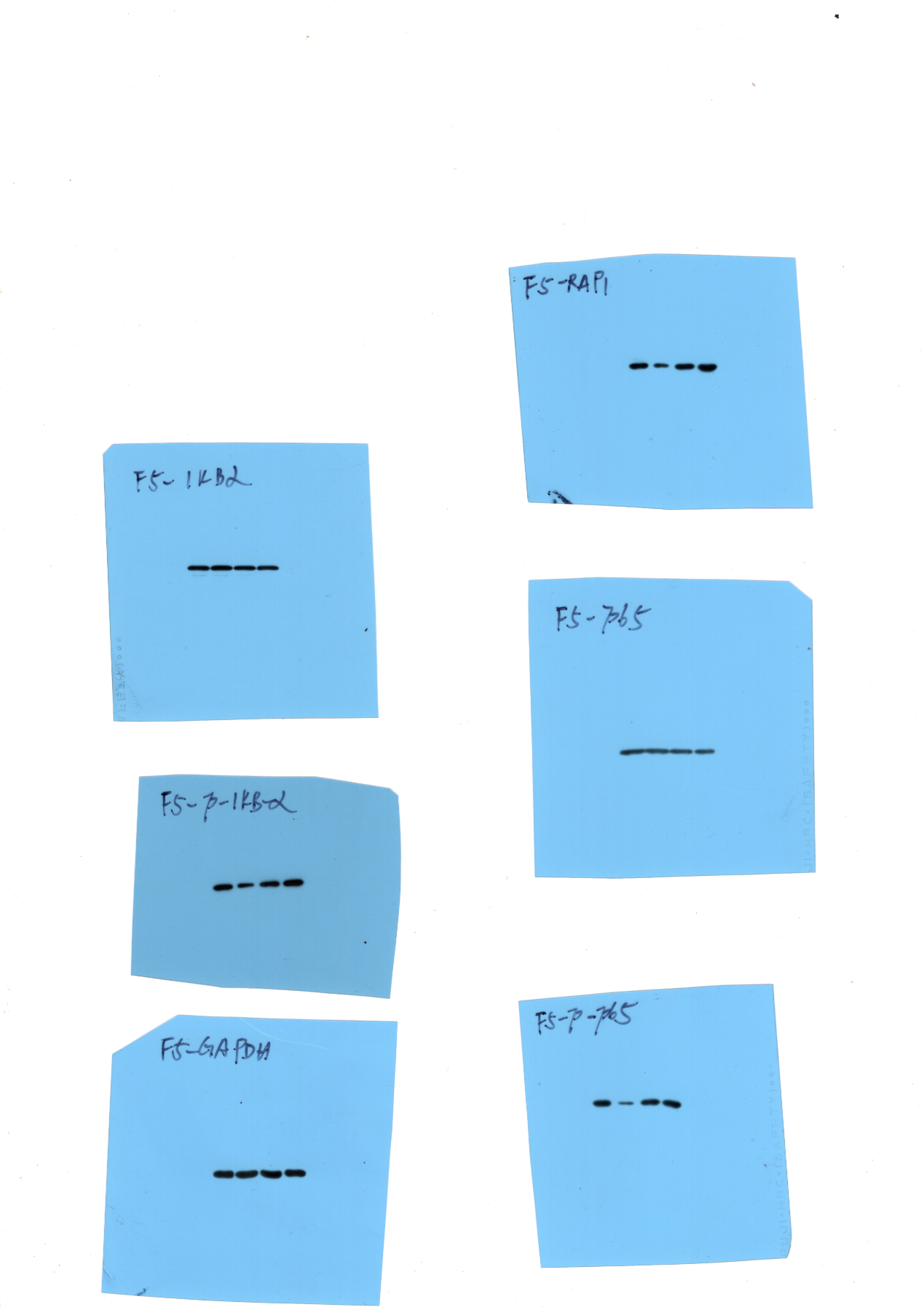

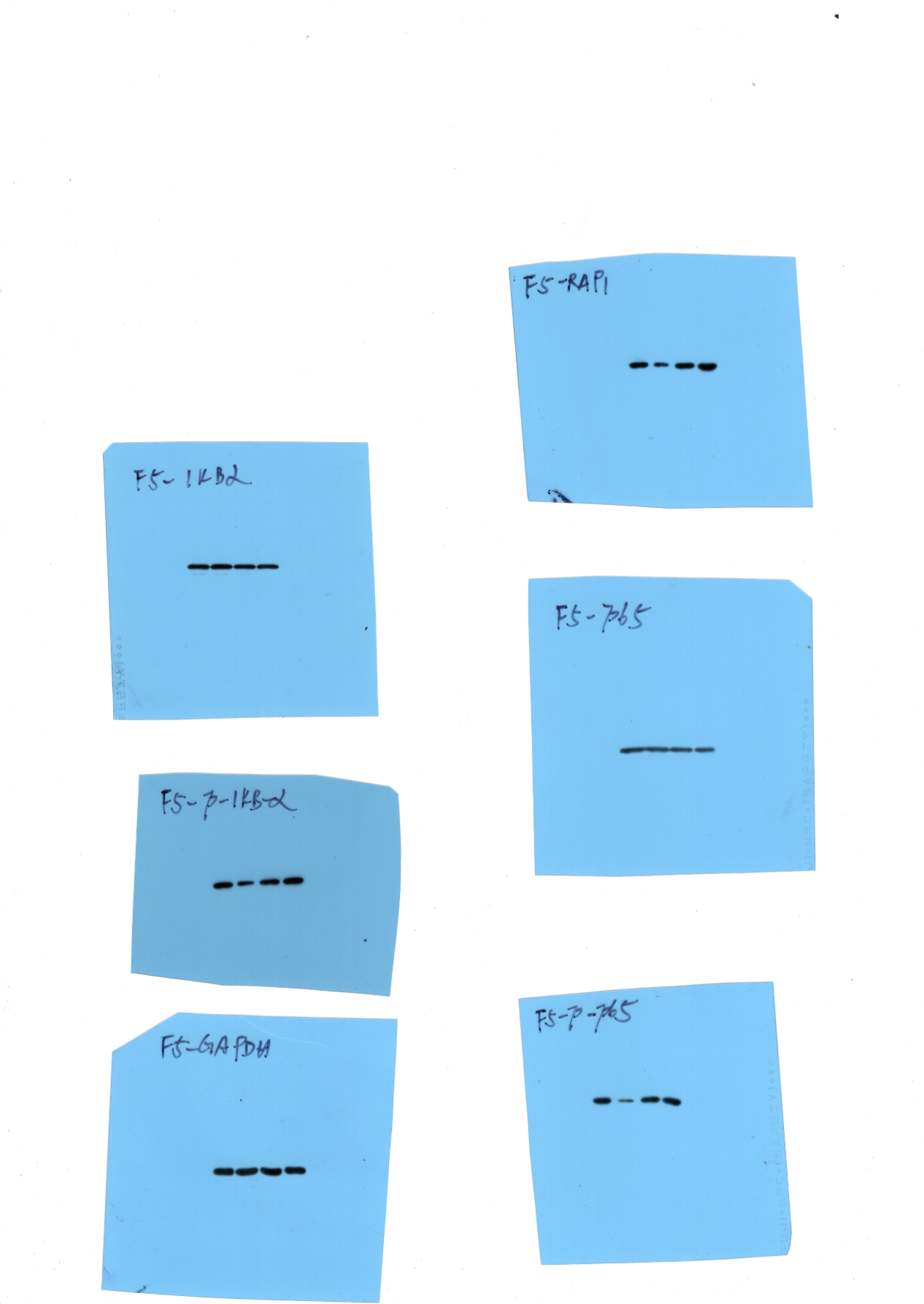
RAP1 (21kDa) IkBα（39 kDa） p- IkBα(40 kDa)


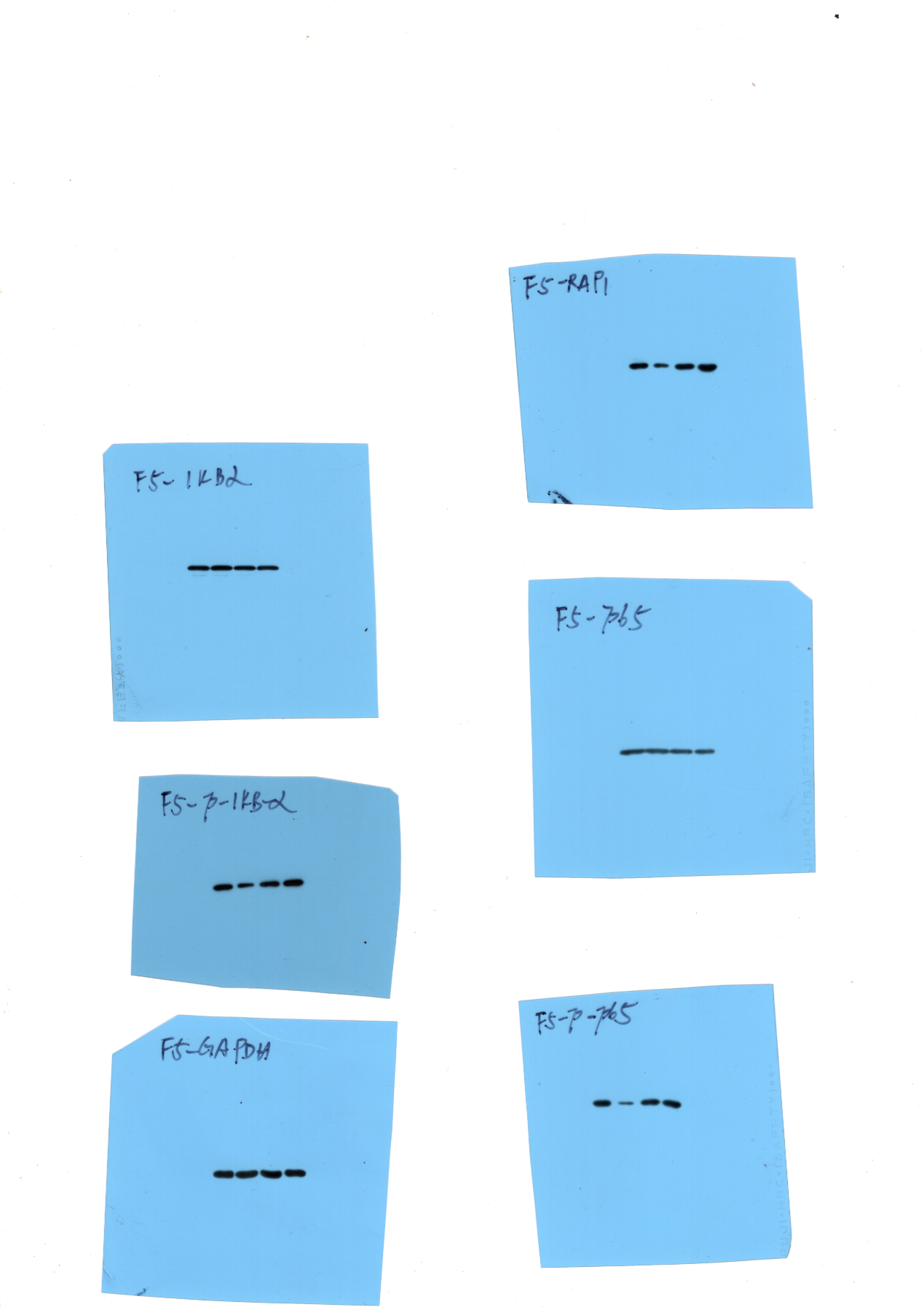


NF-kB p65 (65kDa) p-NF-kB p65 (65kDa) GAPDH（37 kDa）

**Figure 5f**


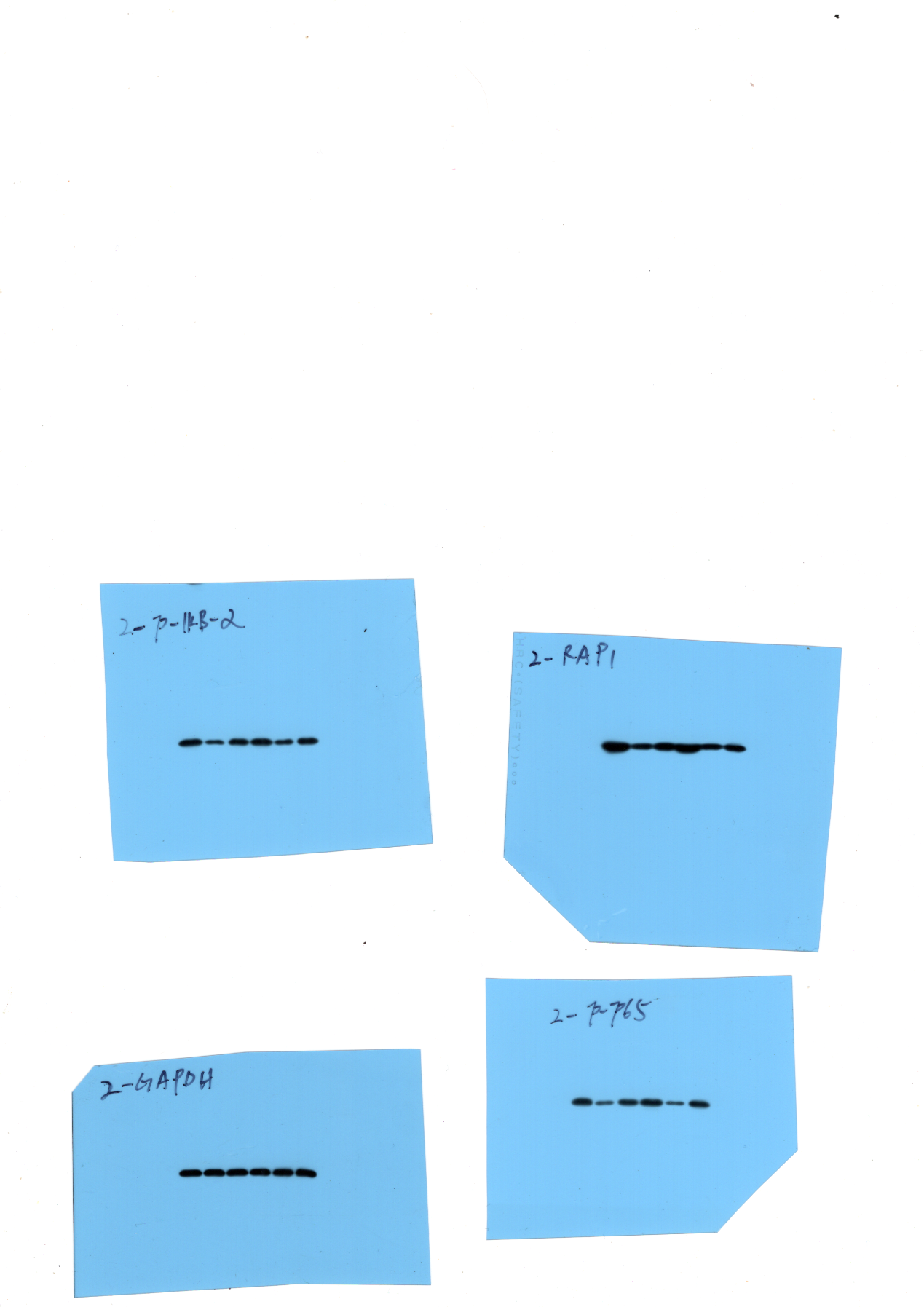


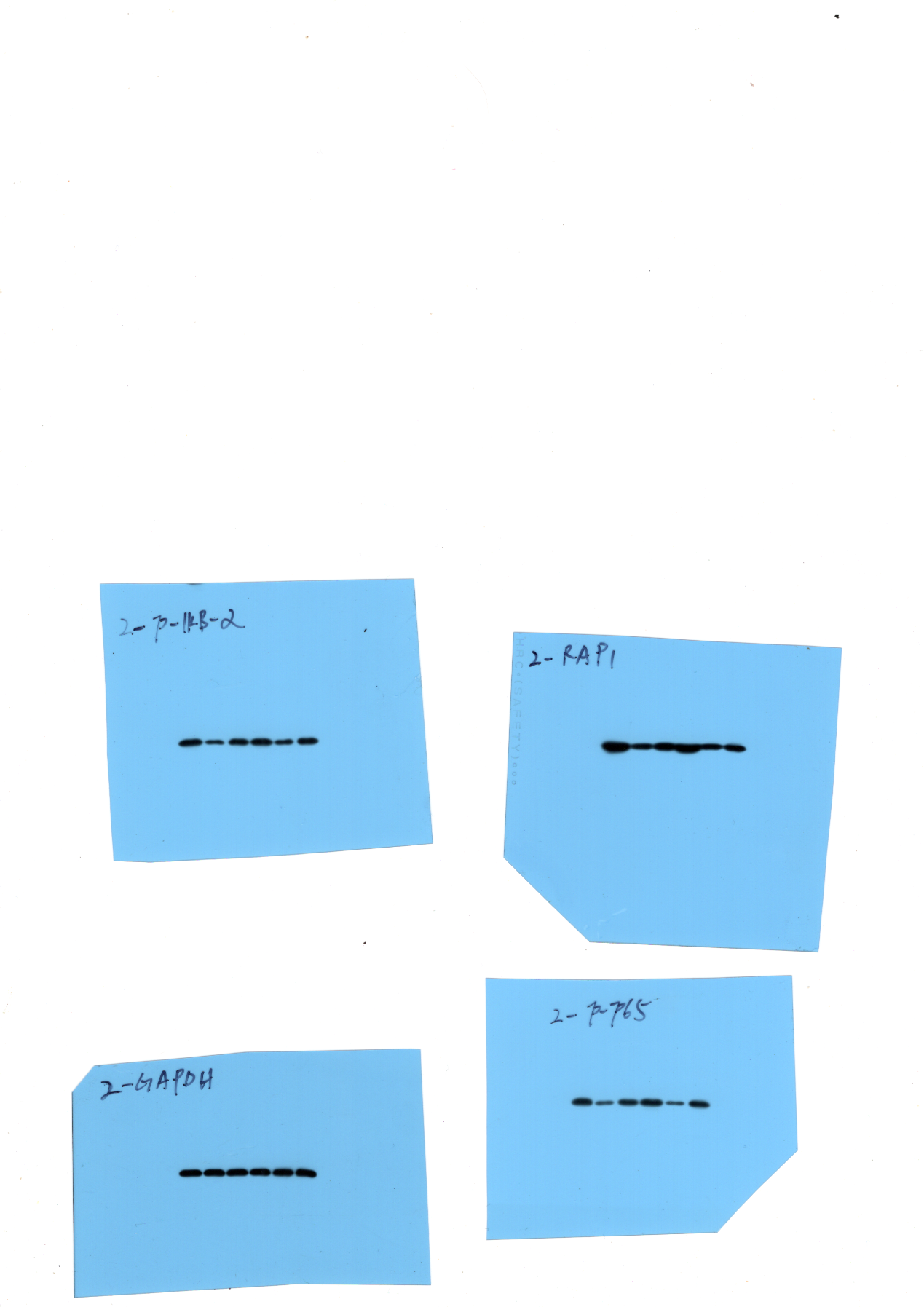


RAP1 (21kDa) p- IkBα(40 kDa)


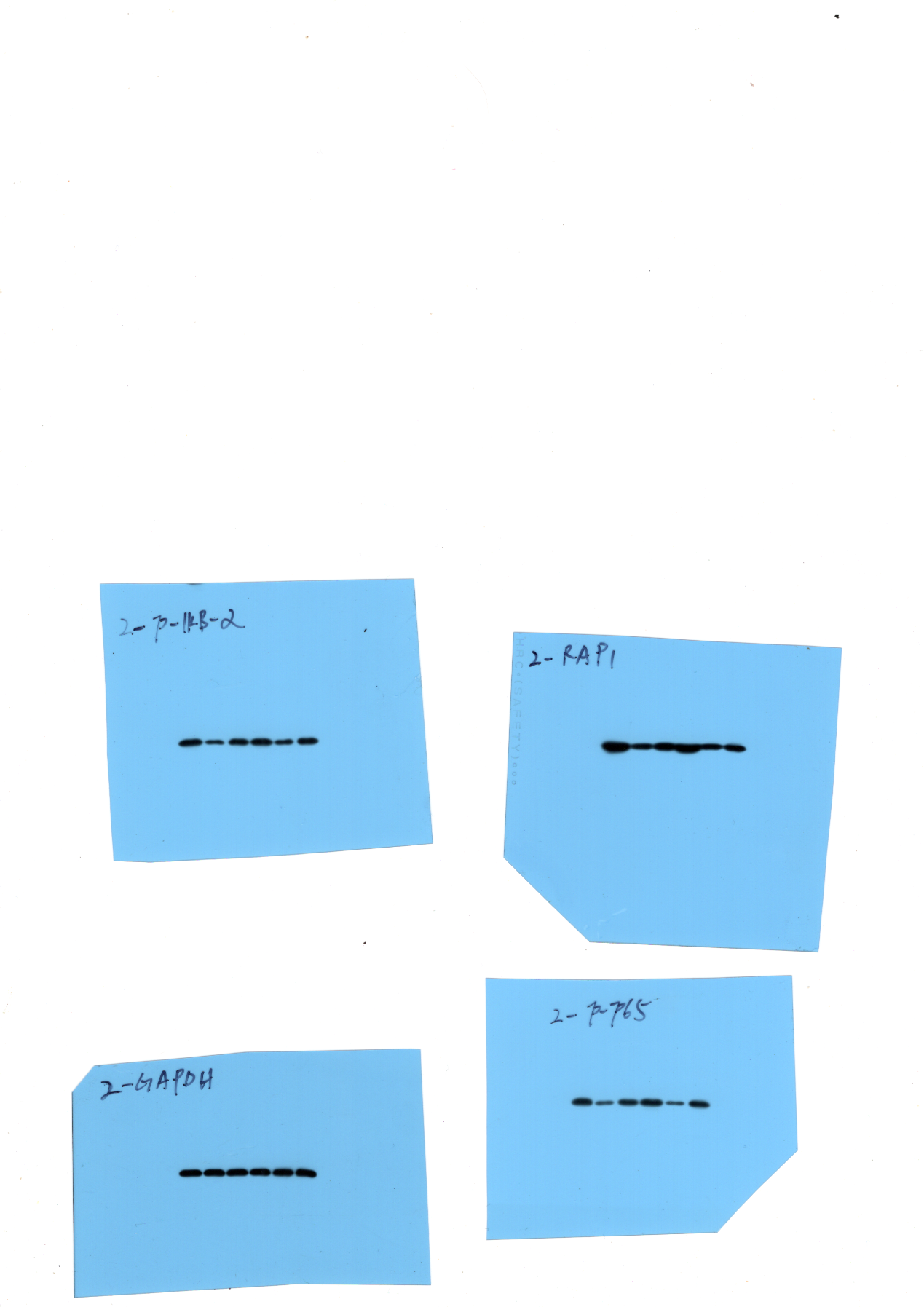

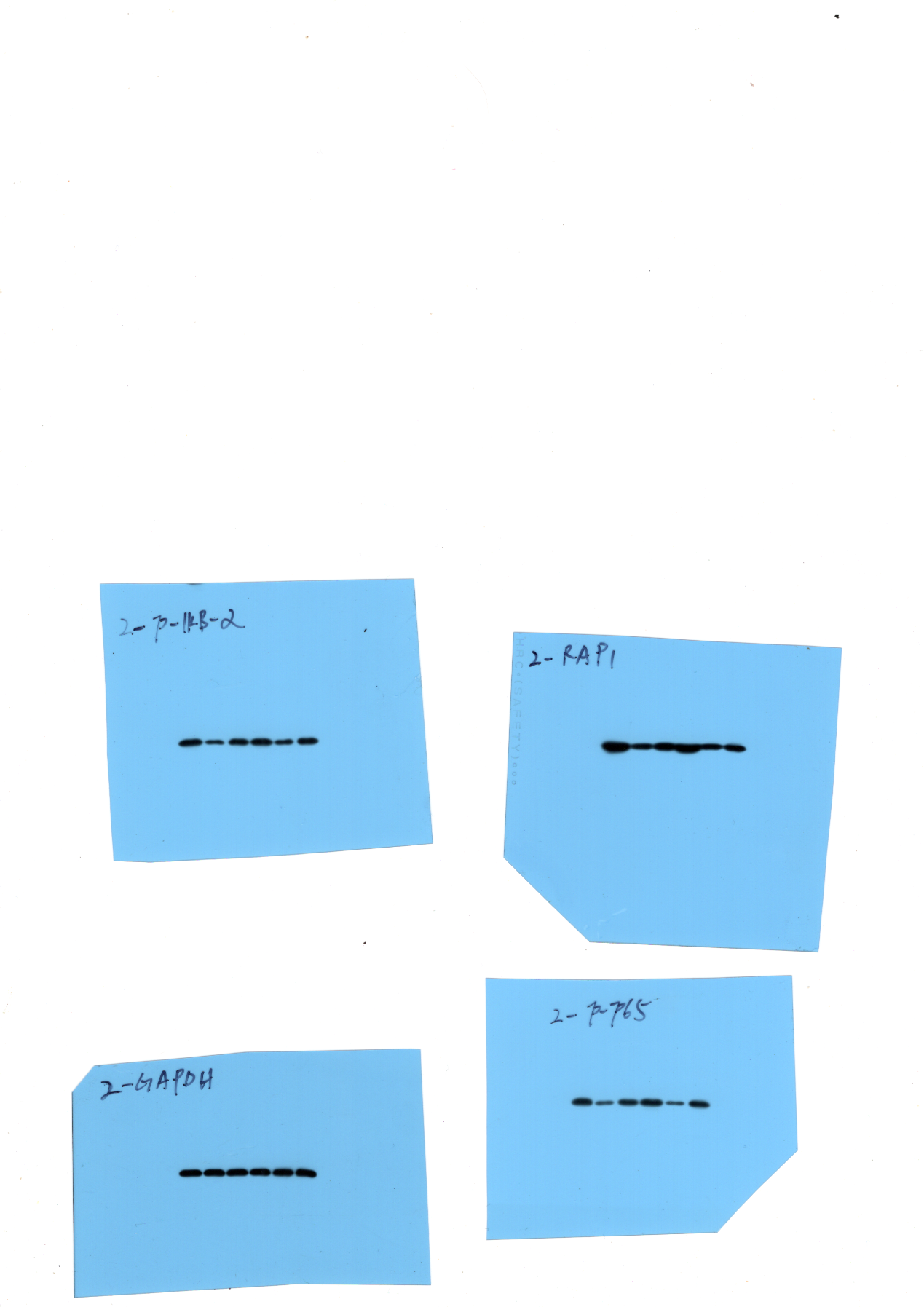


p-NF-kB p65 (65 kDa) GAPDH (37 kDa)

**Figure 5g A549/DDP cell line**




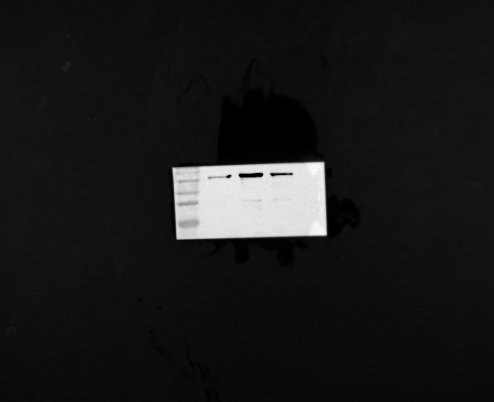






Nuclear p65(65 kDa) H3 (15kDa) Cytoplasmic p65 (65 kDa) GAPDH (37 kDa)

**Figure 5g SKMES/DDP**






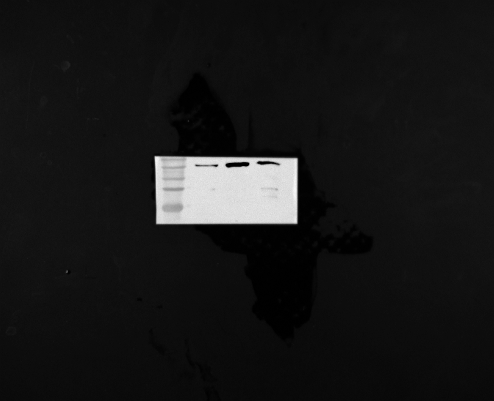




Nuclear p65(65 kDa) H3 (15kDa) Cytoplasmic p65 (65 kDa) GAPDH (37 kDa)



**Figure S4a A549**









RAP1 (21kDa) p-IkBα(40 kDa) p-NF-kB p65 (Ser 536) (65 kDa) GAPDH(37 kDa)

**Figure S4a SKMES**












RAP1 (21kDa) p-IkBα(40 kDa) p-NF-kB p65 (Ser 536) (65 kDa) GAPDH(37 kDa)

**Figure S4e A549**










Nuclear p65(65 kDa) H3 (15kDa) Cytoplasmic p65 (65 kDa) GAPDH (37 kDa)

**Figure S4e SKMES**












Nuclear p65(65 kDa) H3 (15kDa) Cytoplasmic p65 (65 kDa) GAPDH (37 kDa)
